# The effects of yoga compared to active and inactive controls on physical function and health related quality of life in older adults- systematic review and meta-analysis of randomised controlled trials

**DOI:** 10.1186/s12966-019-0789-2

**Published:** 2019-04-05

**Authors:** Divya Sivaramakrishnan, Claire Fitzsimons, Paul Kelly, Kim Ludwig, Nanette Mutrie, David H. Saunders, Graham Baker

**Affiliations:** 1 Physical Activity for Health Research Centre (PAHRC), University of Edinburgh, St Leonard’s Land, Edinburgh, EH8 8AQ UK; 2 Institute for Clinical Exercise and Health Science, University of the West of Scotland, Lanarkshire Campus, South Lanarkshire, G72 0LH UK

**Keywords:** Physical activity, Strength, Balance, Flexibility, Wellbeing, Depression, Sleep, Vitality

## Abstract

**Background:**

Yoga has been recommended as a muscle strengthening and balance activity in national and global physical activity guidelines. However, the evidence base establishing the effectiveness of yoga in improving physical function and health related quality of life (HRQoL) in an older adult population not recruited on the basis of any specific disease or condition, has not been systematically reviewed. The objective of this study was to synthesise existing evidence on the effects of yoga on physical function and HRQoL in older adults not characterised by any specific clinical condition.

**Methods:**

The following databases were systematically searched in September 2017: MEDLINE, PsycInfo, CINAHL Plus, Scopus, Web of Science, Cochrane Library, EMBASE, SPORTDiscus, AMED and ProQuest Dissertations & Theses Global. Study inclusion criteria: Older adult participants with mean age of 60 years and above, not recruited on the basis of any specific disease or condition; yoga intervention compared with inactive controls (example: wait-list control, education booklets) or active controls (example: walking, chair aerobics); physical function and HRQoL outcomes; and randomised/cluster randomised controlled trials published in English. A vote counting analysis and meta-analysis with standardised effect sizes (Hedges’ g) computed using random effects models were conducted.

**Results:**

A total of 27 records from 22 RCTs were included (17 RCTs assessed physical function and 20 assessed HRQoL). The meta-analysis revealed significant effects (5% level of significance) favouring the yoga group for the following physical function outcomes compared with inactive controls: balance (effect size (ES) = 0.7), lower body flexibility (ES = 0.5), lower limb strength (ES = 0.45); compared with active controls: lower limb strength (ES = 0.49), lower body flexibility (ES = 0.28). For HRQoL, significant effects favouring yoga were found compared to inactive controls for: depression (ES = 0.64), perceived mental health (ES = 0.6), perceived physical health (ES = 0.61), sleep quality (ES = 0.65), and vitality (ES = 0.31); compared to active controls: depression (ES = 0.54).

**Conclusion:**

This review is the first to compare the effects of yoga with active and inactive controls in older adults not characterised by a specific clinical condition. Results indicate that yoga interventions improve multiple physical function and HRQoL outcomes in this population compared to both control conditions. This study provides robust evidence for promoting yoga in physical activity guidelines for older adults as a multimodal activity that improves aspects of fitness like strength, balance and flexibility, as well as mental wellbeing.

**Trial registration:**

PROSPERO registration number: CRD42016038052.

**Electronic supplementary material:**

The online version of this article (10.1186/s12966-019-0789-2) contains supplementary material, which is available to authorized users.

## Background

The World Health Organization’s physical activity (PA) recommendations for older adults (aged 65 years and over) include aerobic, muscle strengthening and balance components [1]. Physical activity levels worldwide decrease with age [2], and the percentage of older adults meeting these recommendations remains low. The United Kingdom (UK) PA guidelines for this age group include the accumulation of at least 150 min of moderate intensity activity or 75 min of vigorous activity per week (MVPA guidelines), as well as activities to improve muscle strength, and balance and coordination on at least two days a week [3]. Thirty-one percent of adults aged 65–74 years and 54% of adults aged 75+ years in England (2015–2016) [4], and 53% of men and 66% of women aged 65 years and over in Scotland (2012–2014) [5], did not meet the MVPA guidelines. The balance guidelines were met by 19% of older men and 12% of older women in Scotland [6]; and only 14% of men and 12% of women in the 65–74 age-group, and 9 % of men and 4 % of women over 75 years met the muscle strength guidelines [6]. Accordingly, the World Health Organization identifies older adults as a strategic priority area for the promotion of physical activity [7].

Yoga is an ancient practice and a way of life that originated in India, and includes the practice of postures, regulated breathing and meditation [8]. It is a mode of activity found to have multiple benefits for older adults [9–11]. Previous systematic reviews have provided evidence on the beneficial effects of yoga in older adults in terms of promoting cardiovascular health [12], balance and mobility [10], alleviating depression and improving quality of sleep [9]. A recent systematic review and meta-analysis by Tulloch et al. [13] found that yoga had a medium effect on health related quality of life (HRQoL), and a small effect on mental wellbeing in people aged 60+ years. In this review, HRQoL was measured by physical component summary scales, and mental wellbeing was assessed by mental component summary scales from questionnaires like SF-36 and WHOQOL. However, HRQoL has been described as a concept encompassing several aspects of overall quality of life that can be clearly shown to affect health [14], including anxiety, stress, depression, vitality, social health and sleep [15], which were not assessed in the review.

Physical function is another relevant outcome for the older adult population and includes aspects such as cardio-respiratory fitness, muscular strength, flexibility and balance [16, 17]. Benefits of performing muscle strength activities in older adults include the offsetting of age-related muscle loss (sarcopenia), enhanced functional performance, improved bone mineral density (BMD), and prevention of falls [18, 19]. Whilst yoga has been specifically recommended as a muscle strengthening activity as part of several national PA guidelines including the UK and United States (US) [20, 21], there have been no previous attempts to synthesise the evidence base to support this recommendation for the older adult population. Patel et al. [11] studied the effects of yoga on some physical function and HRQoL outcomes in older adults from randomised controlled trials (RCT) published between 1950 and 2010. Results of the meta-analysis showed that yoga may be significantly better than controls in improving self-rated health status and aerobic fitness, but no significant differences were found for depression. However, the narrative and quantitative analysis in the Patel et al. review [11] combined data in which yoga was compared with active (example: walking, Tai chi, stretching exercises) and inactive controls (example: usual care, socialisation, education group), making it difficult to draw conclusions on whether any true effects (statistically significant) of yoga compared to other exercise programmes exist, and the strength (magnitude) of these effects.

Tulloch et al. [13] and Patel et al. [11] included studies involving older participants with clinical conditions. Other systematic reviews have focused on yoga in specific clinical groups such as cancer [22], Type 2 Diabetes [23, 24] and rheumatic diseases [25], and found some evidence that yoga has beneficial effects on physiological, physical function and psychosocial outcomes in these populations. Results from studies which only recruited participants with specific diseases or conditions cannot be generalised to all older adults. The yoga interventions used in studies involving clinical populations may have been specially developed to address particular symptoms (example: dyspnea related distress in older adults with chronic obstructive pulmonary disease [26]). It is also difficult to disentangle the effects of yoga when data from heterogeneous groups with different clinical conditions are merged in a review.

Therefore, the present systematic review aims to address limitations in previous reviews and expand on existing evidence in three ways: i) including a comprehensive list of physical function and HRQoL outcome measures; ii) comparing yoga against distinct active and inactive controls so that the relative benefits of yoga can be assessed; and iii) reviewing the effectiveness of yoga in studies where older adult participants were not recruited on the basis of a specific disease or condition. The objective of this review was to assess the effectiveness of yoga compared to active and inactive controls on physical function and HRQoL in older adults not characterised by a specific clinical condition, based on randomised/cluster randomised controlled trials.

## Methods

The review was conducted in accordance with Preferred Reporting Items for Systematic Reviews and Meta-Analyses (PRISMA) guidelines [27], and recommendations of the Cochrane collaboration [28]. The protocol was developed in advance of the study and registered on PROSPERO (Registration number: CRD42016038052).

### Search and selection criteria

The inclusion and exclusion criteria for studies were as follows: (i) Participants: older adults defined as mean age 60 years and above, not recruited on the basis of a specific disease or condition were included; (ii) Intervention and comparison: studies comparing yoga interventions with active and inactive controls were included. Studies in which yoga was specified as a control condition or where yoga was combined with other practices or exercise forms were excluded; (iii) Outcomes: only studies reporting physical function and/or HRQoL outcomes were included; (iv) Study type: studies with a randomised (including cluster randomised) controlled study design published in English were included.

A mean age of 60 years and above was a criterion for inclusion. The retirement age in countries like India and China is 60 years [29, 30], and the United Nations defines older persons as those aged 60 years or over [31]. To accommodate these definitions of old age, the age criterion for inclusion in this review was set as a mean of 60+ years. Another criterion was the inclusion of participants who were not recruited based on a disease or condition, and this meant excluding studies in which participants were recruited specifically if they had a particular disease or clinical condition. However, studies with frail, inactive older adults, and those with poor balance were included in the review.

Studies with yoga as a control group were excluded from the review (*n* = 6) [32–37]. In these studies, the yoga group was used to control for aspects such as social stimulation and attention from trainers, without producing an aerobic response. The reporting for the controls was not rigorous, and the yoga programmes were not described in detail. Some studies dated back to 1989, making it difficult to procure the necessary data for them.

### Search and screening

Database searches were conducted in September 2017. The following databases were searched (from inception till September 2017): Medline, PsycInfo, CINAHL Plus, Scopus, Web of Science, Cochrane Library, Embase, SPORTDiscus, AMED, ProQuest Dissertations & Theses Global. The search was conducted using key words related to “yoga” and “older adults”. A detailed list of the search terms used is presented in the supplementary section (Additional file 1). The outcome and study type criteria were applied at the screening stage. The reference lists of included studies were also checked for additional relevant studies [38].

Screening was carried out in three stages using reference management software (EndNote X7.2.1). First, a preliminary title and abstract screening was performed by one researcher (DiS) where duplicates and obviously irrelevant studies were removed. Five percent of the search results were cross-checked by another researcher (KL). Second, titles and abstracts of all studies were screened by two researchers (DiS, KL) with studies categorised as “Yes” (satisfied eligibility criteria), “No” (did not satisfy eligibility criteria) and “Maybe” (uncertain, and need further scrutiny). Finally, full texts of studies in the “Yes” and “Maybe” categories were screened in further detail by two researchers (DiS, KL). Disagreements were resolved by a third researcher (CF or GB).

### Data extraction

A custom data extraction form for descriptive characteristics (Additional file 2) was developed and piloted by three researchers (DiS, GB, CF). Descriptive data were extracted for all included studies by one researcher (DiS), and 33% of these were cross-checked by another researcher (GB or KL). Outcome data were extracted by one researcher (DiS), and 100% cross-checked by another researcher (KL). Discrepancies were resolved through discussions among the researchers (DiS, KL). Authors of studies for which outcome data were not available were contacted and requested to provide the data, and were asked for clarifications if required. One study only reported median, minimum and maximum values for outcome variables [39]. Means and standard deviation were imputed from these data [40–42], and the study was included in the meta-analysis.

### Quality assessment

Risk of bias was assessed independently by two researchers (DiS, KL) using the Cochrane risk of bias tool [43]. The following domains were assessed for physical function and HRQoL outcomes separately: selection bias (random sequence generation, allocation concealment), detection bias (blinding of outcome assessment), attrition bias (incomplete outcome data), reporting bias (selective reporting), and other bias (sample selection bias [44–47], contamination bias [45, 46], compliance bias [46] and response bias [48]). Performance bias (blinding of participants and personnel) was not assessed as it is impossible to blind participants and personnel in a yoga intervention study. Under each domain, studies were classified as low, high or unclear risk of bias. Discrepancies were resolved through discussion between the two researchers.

### Analysis

For the physical function and HRQoL variables, separate analyses comparing yoga with active and inactive groups were conducted. Other sub-group analyses such as types of yoga and gender were not explored. Though different yoga types have been used in the included studies, there is similarity between types in terms of the structure and postures followed and hence, it was not considered appropriate to compare them. Further, the requisite outcome data were not readily available by gender for a majority of studies.

#### Vote-counting

As a preliminary analysis, a ‘vote counting’ approach was adopted [49], where three categories were created for each outcome: statistically significant (as reported by authors) positive effects favouring the yoga group, statistically significant negative effects (i.e., favouring the control group), and no significant difference between groups. For every outcome, effects of yoga was based on the category with the highest number of vote counts. For example, for strength, if the majority of studies found significant positive results favouring yoga, then yoga was considered to have a positive effect [49]. Vote-counting has been critiqued as crude and flawed as it does not give due weight to sample size and effect size (ES). However, when used in conjunction with a meta-analysis, the method can provide a comprehensive understanding of the studies and outcomes included, and the effects of the intervention [49]. The vote-counting approach helped create a catalogue of all results from every study included in the systematic review, providing a foundational structure based on which the data for the meta-analysis were generated. The vote counting analysis included all outcomes assessed by more than one study, and the included outcomes are presented in Table 1.

**Table 1:** List of outcomes included and not included in vote-counting and meta-analysis

Analysis	Active/inactive control group	Physical functioning outcomes	HRQoL outcomes
Vote counting analysis	Yoga vs inactive controls	Body composition measures (body mass index (BMI), body weight, body fat percentage, waist circumference), cardio-respiratory fitness, strength (lower and upper limb strength, hand grip strength), flexibility (lower and upper body flexibility, range of motion (ROM)), mobility, walking speed, balance, fall frequency	Anxiety, depression, perceived physical health, perceived mental health, general health and wellbeing (subscale from SF-12 and SF-36), vitality, quality of life, social health, sleep quality, stress, fear of falls, balance confidence
	Yoga vs active controls	Strength (lower and upper limb strength), flexibility (lower and upper body flexibility), mobility, walking speed, balance, fall frequency	Anxiety, depression, perceived physical health, perceived mental health, vitality, stress
Meta-analysis	Yoga vs inactive controls	Body composition (BMI, body weight and body fat percentage), balance, lower body flexibility, upper body flexibility, walking speed, lower limb strength	Depression, fear of falls, perceived mental health, perceived physical health, sleep quality, social health, vitality
	Yoga vs active controls	Balance, lower body flexibility, lower limb strength, mobility, walking speed	Anxiety, depression, perceived mental health
Not included in vote-counting or meta-analysis (measured by just one study)	Yoga vs inactive controls		Anger [63], self-control [63], fatigue [64], motivational factors to exercise [55], pain [71], mood [64], hope [65], medication usage [66], capacity for self-care [66], self-efficacy (general, and for chronic disease) [63]
	Yoga vs active controls	Cardio-respiratory fitness [72]	Anger [63], self-control [63], fatigue [64], motivational factors to exercise [55], pain [71], mood [64], social health [64], general health and well-being [64], quality of life [71], balance confidence [67], fear of falls [67], self-efficacy (general, and for chronic disease) [63]

#### Meta-analysis

For outcomes where quantitative data from three or more studies were available, a meta-analysis was conducted using the Comprehensive Meta-Analysis Version 3, Professional software. The outcomes included in the meta-analysis are presented in Table 1. Some studies used more than one test or instrument to measure an outcome. Since only one of these could be included in the meta-analysis, the test most commonly reported by the included studies was chosen. For balance, only functional assessments [50] such as one leg stand test, Berg balance scale, standing balance tests from the Short Physical Performance Battery, and Performance Oriented Mobility Assessment (POMA) were included in the meta-analysis. Objective measures like static and dynamic posturography [50] were not included due to the lack of a composite index and difficulties in interpreting the data. A random effects model was used as it better models data from heterogeneous populations [51]. Data at pre-intervention and immediately following the intervention were analysed, and effect sizes were calculated based on change (post minus pre) scores. Since various different instruments and units were used by studies to measure outcomes, calculation of mean differences was not possible, and standardised mean differences (SMD) were computed instead [51]. Hedges’ g statistic was used to compute effect sizes, and Forest plots were created with 95% confidence intervals (CI). Effect sizes were categorised as small (0.2 to 0.5), moderate (0.5 to 0.8) and large (> 0.8) using Cohen’s categories [52]. Statistical heterogeneity between studies was assessed using the I^2^ statistic. Heterogeneity was categorised as low (I^2^ = 0 to 40%), moderate (I^2^ = 30 to 60%), substantial (I^2^ = 50 to 90%) and considerable (I^2^ = 75 to 100%) [51].

One study had two yoga intervention groups and one control group [53]. Both yoga groups were included in the meta-analysis, each one compared with half the number of participants in the control group [54]. Four studies [55–58] had one yoga intervention group and two control groups. In these cases the result was included twice in the meta-analysis with half the number of participants for the yoga group each time [54]. Following this, two sensitivity analyses were also conducted: (i) comparing the full yoga intervention arm and the first control group, and (ii) comparing the full yoga intervention arm and the second control group. Five included studies [53, 57, 59–61] used cluster randomisation, and an iteration of the meta-analysis was run after adjusting the number of participants in the studies to account for this (adjusted sample size = original sample size /design effect, where design effect = 1+ (Average cluster size - 1)*Intracluster Correlation Coefficient; calculations presented in Additional file 3) [54]. Four studies [53, 59–61] had the requisite data for cluster randomisation adjustment, and one study [57] was removed from this analysis due to lack of data. There were insufficient studies (less than 10) in the meta-analyses to test for publication bias using funnel plots [62].

## Results

**Fig. 1:**
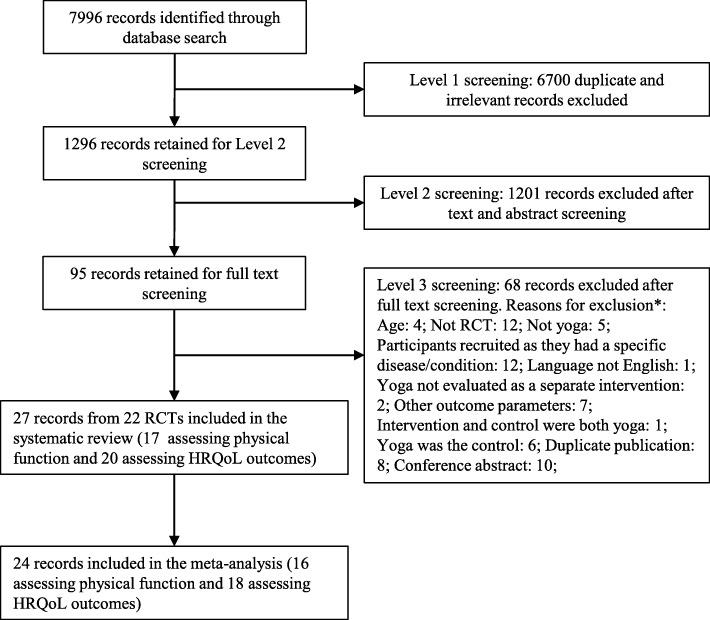
Flow of studies through the review. Legend: *Studies could have been excluded for more than one reason

7996 records were identified through the data searches, and after the three stages of screening, 27 records from 22 RCTs (Fig. 1) were included in the systematic review. Seventeen RCTs with 967 participants assessed physical function, and 20 RCTs with 1567 participants assessed HRQoL.

### Study and participant characteristics (Table 2)

Ten studies were from the USA [55, 56, 63–70], four from Australia [39, 71–73], two from Taiwan [53, 59], two from India [57, 74], and one each from Brazil [75], Iran [76], UK [77] and Portugal [78]. The number of participants in studies ranged from 18 to 410, and the mean size was 77 ± 84.

**Table 2:** Participant, intervention and study characteristics

Study id Country	Participants (number, mean age (SD))	Intervention group (type, frequency, session duration, length of intervention)	Control group(s)	Outcome measures
Bezerra (2014) [75] Brazil	*N* = 36 Mean age (SD): yoga group = 63.1 (13.3); control group = 61.0 (6.9) years.	Yoga, 3 times a week, 65 min per session, 12 weeks	1. IC: Control group	Physical function: Body weight
Bonura (2014) [63] USA	*N* = 98 Mean age (SD): 77.04 (7.28) years	Chair yoga, 1 session a week, 45 min per session, 24 weeks	1. AC: Chair exercise 2. IC: Wait-list control	HRQoL: Anger, anxiety, depression, well-being, general self-efficacy, self-efficacy for daily living, self-control
Bethany (2005) [55] USA	*N* = 42 Mean age (SD): 83.14 (4.84) years	Chair yoga, 3 times a week, 30 min per session, 6 weeks	1. AC: Chair aerobics 2. AC: Walking programme 3. IC: Game playing	HRQoL: Stress frequency, stress severity, state anxiety, depression, motivational factors to exercise
Chen (2008) [53] Taiwan	*N* = 176 Mean age (SD): Complete Silver Yoga = 65.81 (4.34); Shortened Silver Yoga = 68.08 (6.32); control group = 72.42 (6.04) years	1. Complete Silver Yoga with meditation, 3 times a week, 70 min per session, 24 weeks 2. Shortened Silver Yoga, 3 times a week, 55 min per session, 24 weeks	1. IC: Wait-list control	Physical function: Bodyweight, BMI, body fat percentage, lower body flexibility, upper limb strength, lower limb strength, balance, walking speed, range of motion: flexion and abduction of shoulder and hip joints on both sides
Chen (2009) [61] Taiwan	*N* = 128 Mean age (SD): 69.20 (6.23) years	Silver Yoga, 3 times a week, 70 min per session, 24 weeks	1. IC: Wait-list control	HRQoL: Sleep quality, depression, perceived mental and physical health
Chen (2010)1 [60] Taiwan	*N* = 55 Mean age (SD): 75.40 (6.70) years	Silver Yoga, 3 times a week, 70 min per session, 24 weeks	1. IC: Wait-list control	HRQoL: Sleep quality, depression, perceived mental and physical health
Chen (2010)2 [59] Taiwan	*N* = 55 Mean age (SD): 75.40 (6.70) years	Silver Yoga, 3 times a week, 70 min per session, 24 weeks	1. IC: Wait-listcontrol	Physical function: Body weight, BMI, body fat percentage, cardiopulmonary fitness, upper body flexibility, lower body flexibility and range of motion: flexion and abduction of shoulder and hip joints on both sides, upper limb and lower limb muscle strength, balance, and walking speed
Gothe (2016) [91] USA	*N* = 108 Mean age (SD): yoga group = 62.1 (5.82); control group = 62.0 (5.39) years	Hatha yoga, 3 times a week, 60 min per session, 8 weeks	1. AC: Stretching–strengthening control group	Physical function: Mobility, upper and lower limb strength, upper and lower body flexibility, balance, walking speed
Gothe (2013) [68] USA	*N* = 108 Mean age (SD): yoga group = 62.1 (5.82); control group = 62.0 (5.39) years	Hatha yoga, 3 times a week, 75 min per session, 8 weeks	1. AC: Stretching–strengthening control group	HRQoL: Stress, anxiety
Haber (1983) [70] USA	Centre1 N: 51 Mean age: 69 years Centre1 N: 35 Mean age: 70 years	The Easy Does it Yoga Programme for Older People, 1 session a week, Daily home practice encouraged, 10 weeks	1. IC: Control group (film series or art class)	HRQoL: Self assessed health status, psychological wellbeing
Haber (1988) [66] USA	*N* = 410 Mean age: 75 years	The Easy Does it Yoga Programme for Older People, 3 times a week, 60 min per session, 10 weeks	1. IC: Control group	HRQoL: Self-care, sociability, medication usage
Hariprasad (2013) [74] India	*N* = 87 Mean age (SD): yoga group = 75.74 (6.46); control group = 74.78 (7.35) years	Yoga, daily supervised sessions for 1 month. 1 session per week in the 2nd and 3rd month. Daily home practice following this, 60 min per session, 24 weeks	1. IC: Wait-list control	HRQoL: Perceived physical and mental health, sleep
Krishnamurthy (2007) [92] India	*N* = 50 Mean age (SD): yoga group = 70.1 (8.3); ayurveda = 72.1 (9.0); wait-list = 72.3 (7.4) years	Yoga, 6 times a week, 60 min per session, 24 weeks	1. IC: Ayurveda group (herbal preparation) 2. IC: Wait-list control	Physical function: Balance, mobility
Krishnamurthy (2007)2 [58] India	*N* = 50 Mean age (SD): yoga group = 70.1 (8.3); ayurveda = 72.1 (9.0); wait-list = 72.3 (7.4) years	Yoga, 6 times a week, 60 min per session, 24 weeks	1. IC: Ayurveda group (herbal preparation) 2. IC: Wait-list control	HRQoL: Depression
Leininger (2006) [69] USA	*N* = 82 Mean age (SD): yoga group = 69.6 (6.7); education group = 68.2 (5.4) years	Hatha yoga, 2 supervised sessions a week. Home exercises recommended at least three times a week, 60 min per session, 10 weeks	1. IC: Education control group (on topics of osteoporosis and fitness)	Physical function: Balance, lower limb strength, HRQoL: Balance confidence, vitality
Manjunath (2005) [57] India	*N* = 50 Mean age (SD): yoga group = 70.1 (8.3); ayurveda = 72.1 (9.0); wait-list = 72.3 (7.4) years	Yoga training, 6 times a week, 60 min per session, 24 weeks	1. IC: Ayurveda (herbal preparation) 2. IC: Wait-list control	HRQoL: Sleep quality
Marques (2017) [78] Portugal	*N* = 25 Mean age (SD): 83.16 (7.4) years	Chair based yoga, 2 to 3 times a week, 50 min per session, 28 weeks	1. IC: Control group given education booklet	Physical function: Cardio-respiratory fitness, mobility, upper body flexibility HRQoL: Stress, perceived mental health
Morris (2008) [67] USA	*N* = 18 Mean age (SD): 76.06 (6.35) years	Hatha yoga, 2 times a week, 60 min per session, 8 weeks	1. AC: Balance training exercise 2. IC: Fall risk awareness	Physical function: Balance, fall frequency HRQoL: Fear of falls, balance confidence
Ni (2014) [56] USA	*N* = 39 Mean age (SD): 74.15 (6.99) years	Balance yoga programme, 2 times a week, 60 min per session, 12 weeks	1. AC: Tai Chi 2. AC: Standard balance programme	Physical function: Mobility, balance, walking speed
Nick (2016) [76] Iran	*N* = 39 Mean age (SD): yoga group = 68 (4.87); control group = 68.79 (4.81) years	Hatha yoga, 2 times per week, 60 min per session, 8 weeks	1. IC: Control group	Physical function: Balance HRQoL: Fear of falls
Noradechanunt (2017) [72] Australia	*N* = 33 Mean age (SD): 67.7 (6.7) years	Thai Yoga, 2 supervised session a week, 80 min per session. Home practice on alternate days for 20 min, 12 weeks	1. AC: Tai Chi 2. IC: Control group	Physical function: Lower and upper limb strength, lower and upper body flexibility, mobility HRQoL: Perceived physical and mental health, depression
Oken (2006) [64] USA	*N* = 118 Mean age (SD): yoga group = 71.5 (4.9); exercise group = 73.6 (5.1); wait-list = 71.2 (4.4) years	Iyengar yoga, 1 class a week with home practice, 90 min per session, 24 weeks	1. AC: Walking group 2. IC: Wait-list control	Physical function: Lower body flexibility, lower limb strength, balance, walking speed HRQoL: Mood, fatigue, depression, perceived physical and mental health, pain, general health and well-being, social functioning, vitality
Saravanakumar (2014) [71] Australia	*N* = 33 Mean age (SD): 83.8 (7.9) years	Yoga, 2 times a week, 30 min per session, 14 weeks	1. AC: Tai Chi 2. IC: Usual care	Physical function: Balance, fall incidence, HRQoL: Pain, quality of life.
Tew (2017) [77] UK	*N* = 47 Mean age (SD): 74.8 (7.2) years	British Wheel of Yoga Gentle Years Yoga programme, 10 sessions during a 12-week period, 75 min. Home practice encouraged for 10–20 min on most days,12 weeks	1. IC: Wait-list control	Physical function: Body weight, BMI, waist circumference, lower limb strength, upper and lower body flexibility, balance, walking speed HRQoL: Perceived mental health, quality of life
Tiedemann (2013) [73] Australia	*N* = 52 Mean age (SD): 68 years (7.1) years	Iyengar yoga, 2 session a week, 60 min. Home practice 2 days a week for 10–20 min, 12 weeks	1. IC: Control group given fall prevention education booklet	Physical function: Balance, lower limb strength, walking speed HRQoL: Fear of falls
Vogler (2011) [39] Australia	*N* = 38 Mean age (SD): 73.21 (8.38) years	Iyengar yoga, 2 times per week, 90 min per session. Home practice 3 days per week for 15–20 min, 8 weeks	1. IC: Wait-list control group	Physical function: Muscle strength, range of motion of the upper extremity, hip flexion, hip extension, hip abduction, and trunk rotation HRQoL: General health and well-being, perceived physical and mental health
Wang (2010) [65] USA	*N* = 18 Mean age (SD): 74.9 (8.4) years	Yoga group, 2 times per week, 60 min per session, 4 weeks	1. IC: Social group	Physical function: Balance, lower limb strength, lower body flexibility HRQoL: Depression, morale, hope, social isolation

HRQoL: health related quality of life; BMI: Body Mass Index; AC: Active control; IC: Inactive control; N: Number of participants analysed in included studies

The mean age of participants in the studies ranged from 61.0 years to 83.8 years. In 15 studies, more than 70% of the participants were female. The attendance rates for class-based yoga sessions ranged from 67 to 100%, and for active controls it was 62 to 91%. Four studies reported adverse events in the yoga group (groin muscle strain [64], fall during yoga session [71], and musculoskeletal pain [73, 77]). Four studies reported that there were no adverse events during the course of the yoga intervention [53, 59, 68, 69].

### Intervention characteristics

Eight types of yoga (Table 3) were used in the studies including Hatha yoga (4 studies) [67–69, 76], chair yoga (3 studies) [55, 63, 78], Iyengar yoga (3 studies) [39, 64, 73], Silver Yoga (2 studies) [53, 59], The Easy Does It Yoga Programme (2 studies) [66, 70], balance yoga programme [56], Thai Yoga [72] and the British Wheel of Yoga (BWY) Gentle Years Yoga programme [77]. Five studies did not mention the type of yoga programme conducted [57, 65, 71, 74, 75]. The most common class structure for the yoga intervention adopted by included studies was a warm up, followed by the main postures, and ending with relaxation, breathing and meditation. Some common postures (used in four or more included studies) are: Cat and cow pose, Tree position, Triangle position, Seated twists, Mountain pose, Warrior 1, Cobra, Chair pose, Eagle or Half eagle, Locust posture, Downward dog, Wind relieving pose, Child’s pose, Standing hands on feet pose, Cow face pose, and Corpse pose (used for relaxation). The length of interventions ranged from four to 28 weeks, the most predominant being 24 weeks (6 studies) [53, 57, 59, 63, 64, 74] followed by 12 weeks (5 studies) [56, 72, 73, 75, 77], and eight weeks (4 studies) [39, 67, 68, 76]. The most common frequency of intervention was two sessions per week (9 studies) [39, 56, 65, 67, 69, 71–73, 76], followed by three sessions per week (6 studies) [53, 55, 59, 66, 68, 75]. Eight studies also encouraged practicing yoga at home in addition to class based sessions [39, 64, 69, 70, 72–74, 77]. Duration of classes ranged from 30 min to 90 min. A 60 min class duration was reported most frequently (9 studies) [56, 57, 65–67, 69, 73, 74, 76]. One study did not report class duration [70]. Inactive controls used in the studies were wait-list control (8 studies) [39, 53, 57, 59, 63, 64, 74, 77], playing games like Dominoes, Chinese Checkers and Scrabble [55], fall risk awareness [67], socialisation [65], education on osteoporosis and fitness [69], fall prevention education booklet [73], herbal preparation [57], telephone counselling [72], film series or art class [70], and usual care where no intervention was provided but participants could continue to use the facilities provided by the residential care centre like bingo, story-telling, exercise classes and gym [71]. Active controls included were Tai Chi (3 studies) [56, 71, 72], chair aerobics/exercise (2 studies) [55, 63], a walking programme (2 studies) [55, 64], balance training (2 studies) [56, 67], and stretching–strengthening exercises [68].

**Table 3:** Types of yoga used in included studies

Types of yoga in included studies (number of studies, total number of participants)	Description
The types of yoga used in studies are similar in structure and postures, and their main features are highlighted below.	
Hatha yoga (4 studies, 247 participants)	Traditional yoga that includes combinations of postures, breathing, and meditation [93].
Chair yoga (3 studies, 165 participants)	This essentially follows a traditional Hatha yoga format, but is modified so that chairs are used during practice to accommodate physical limitations [63].
Iyengar yoga (3 studies, 208 participants)	Created by BKS Iyengar; based on Hatha yoga, but emphasis is on strength, balance, and use of props. Usually involves slow movement and holding poses [93].
Silver Yoga (2 studies, 231 participants)	The programme is based on Hatha yoga and Raja yoga (type of yoga that focuses on concentration and meditative techniques). The programme includes gentle stretching postures to increase range of motion and progressive muscle relaxation. Special consideration given for the physical abilities and tolerance of older adults [94].
Balance yoga programme (1 study, 39 participants)	This programme is based on a study by the authors showing specific muscle utilization patterns during different flow-based yoga poses. The programme has three levels of difficulty, becoming progressively challenging [56].
The Easy Does It Yoga Programme (2 studies, 496 participants)	Yoga programme designed for older adults [66].
Thai Yoga (1 study, 33 participants)	Thai Yoga is similar to the Hatha yoga style. However, it is less strenuous and incorporates postures that are less challenging and easier to perform than those of Hatha yoga [72].
British Wheel of Yoga (BWY) Gentle Years Yoga programme (1 study, 47 participants)	The British Wheel of Yoga (BWY) Gentle Years Yoga programme was developed to cater to the needs of older people with age-related conditions (osteoarthritis, hypertension, dementia, and sensory impairment). Hatha yoga poses were adapted so that older adults with comorbidities and physical limitations could safely participate [77].

### Results of vote counting

The vote-counting tables with all results for both physical function and HRQoL outcomes are presented in the supplementary section (Additional file 4).

#### Physical function

For yoga vs inactive controls, the “favouring yoga” category received most votes for the following outcomes (presented as: number of results where yoga had significantly positive effects compared with control / total number of results): cardio-respiratory fitness (2/3), flexibility (17/23, with lower body flexibility (5/7), ROM (10/13), upper body flexibility (2/3)), and walking speed (3/5). On no occasion did the inactive controls group receive more votes than yoga.

While comparing yoga and active controls, the “no significant difference” category got the highest number of votes for all outcomes.

#### HRQoL

For yoga vs inactive controls, the “favouring yoga” category received most votes for the following outcomes: quality of life (2/3), and sleep quality (3/4). In the yoga vs active controls analysis, the “favouring yoga” category did not receive the highest number of votes for any of the outcomes. The “favouring control” category received no votes for both yoga vs active and yoga vs inactive controls for any HRQoL outcomes.

### Meta-analysis

Sixteen studies assessing physical function and 17 assessing HRQoL variables (from 18 records) were included in the meta-analysis (Table 4). Data used for meta-analysis are attached as supplementary tables (Additional file 5).

**Table 4:** Meta-analysis results- effect sizes and heterogeneity

Outcome	No. of studies	Total number of participants	Effect size		Heterogeneity	
			Hedges’ g (95% CI)	*P*-value	I^2^	*P*-value
Physical function - Yoga vs inactive controls						
Balance	7	265	**0.70 (0.19 to 1.22)**	**0.01**	72.15	0.001
Body composition	4	314	0.16 (−0.06 to 0.38)	0.16	0.00	0.91
Lower body flexibility	7	431	**0.50 (0.3 to 0.69)**	**< 0.001**	0.00	0.88
Lower limb strength	7	485	**0.45 (0.22 to 0.68)**	**< 0.001**	32.70	0.17
Upper body flexibility	4	166	0.28 (−0.02 to 0.58)	0.07	0.00	0.87
Walking speed	5	377	0.38 (−0.02 to 0.78)	0.06	72.69	0.003
Physical function - Yoga vs active controls						
Balance	5	264	0.32 (−0.02 to 0.66)	0.07	34.74	0.18
Lower body flexibility	3	225	**0.28 (0.01 to 0.54)**	**0.04**	0.00	0.59
Lower limb strength	3	225	**0.49 (0.1 to 0.88)**	**0.01**	47.44	0.15
Mobility	3	173	0.31 (−0.25 to 0.87)	0.28	58.73	0.06
Walking speed	3	192	−0.29 (− 0.79 to 0.22)	0.26	57.41	0.07
HRQoL - Yoga vs inactive controls						
Depression	8	450	**0.64 (0.32 to 0.95)**	**< 0.001**	57.09	0.02
Fear of falls	3	104	0.39 (−0.45 to 1.24)	0.36	75.64	0.02
Perceived mental health	9	554	**0.6 (0.33 to 0.87)**	**< 0.001**	54.87	0.02
Perceived physical health	5	400	**0.61 (0.29 to 0.94)**	**< 0.001**	58.55	0.05
Sleep quality	4	353	**0.65 (0.41 to 0.88)**	**< 0.001**	13.06	0.33
Social health	3	225	0.27 (−0.15 to 0.69)	0.2	51.76	0.13
Vitality	3	196	**0.31 (0.03 to 0.59)**	**0.03**	0.00	0.83
HRQoL - Yoga vs active controls						
Anxiety	3	206	0.43 (−0.03 to 0.88)	0.06	50.03	0.11
Depression	4	215	**0.54 (0.25 to 0.83)**	**< 0.001**	8.61	0.36
Perceived mental health	3	183	0.26 (−0.03 to 0.55)	0.08	0.00	0.81

CI: Confidence interval; Significant effect sizes (95% CI) and corresponding p values have been highlighted in bold

#### Physical function

##### Yoga vs inactive controls

**Fig. 2:**
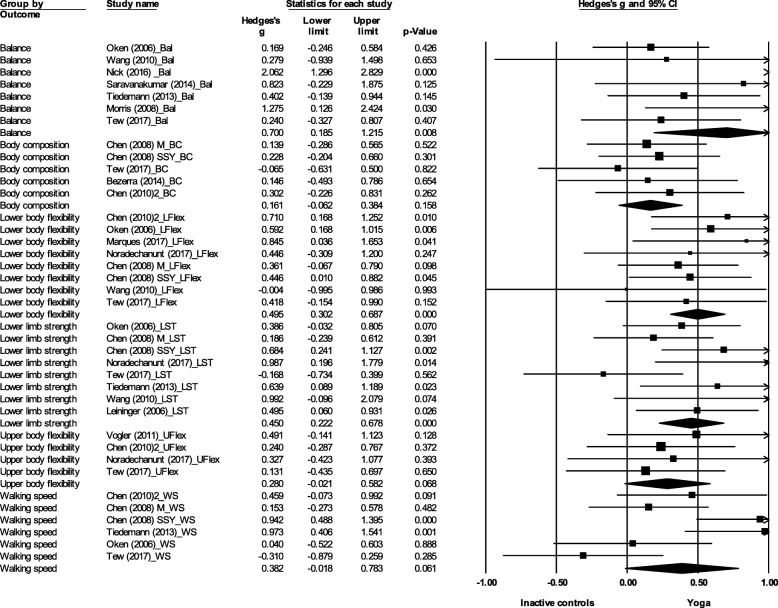
Effect sizes and 95% confidence intervals for yoga compared to inactive controls on physical function outcomes. Legend: Bal: Balance; M: Yoga group with meditation; SSY: Short Silver Yoga; BC: Body composition; LFlex: Lower body flexibility; LST: Lower limb strength; UFlex: Upper body flexibility; WS: Walking speed

Yoga was found to significantly improve balance (ES (Hedges’ g) = 0.7, 95% CI 0.19 to 1.22, *p* = 0.01), lower limb strength (ES = 0.45, 95% CI 0.22 to 0.68, *p* < 0.001), and lower body flexibility (ES = 0.50, 95% CI 0.30 to 0.69, *p* < 0.001) compared to inactive controls (Fig. 2). No significant difference between yoga and inactive controls was found for body composition (ES = 0.16, 95% CI -0.06 to 0.38, *p* = 0.16), upper body flexibility (ES = 0.28, 95% CI -0.02 to 0.58, *p* = 0.07) or walking speed (ES = 0.38, 95% CI -0.02 to 0.78, *p* = 0.06).

##### Yoga vs active controls

**Fig. 3:**
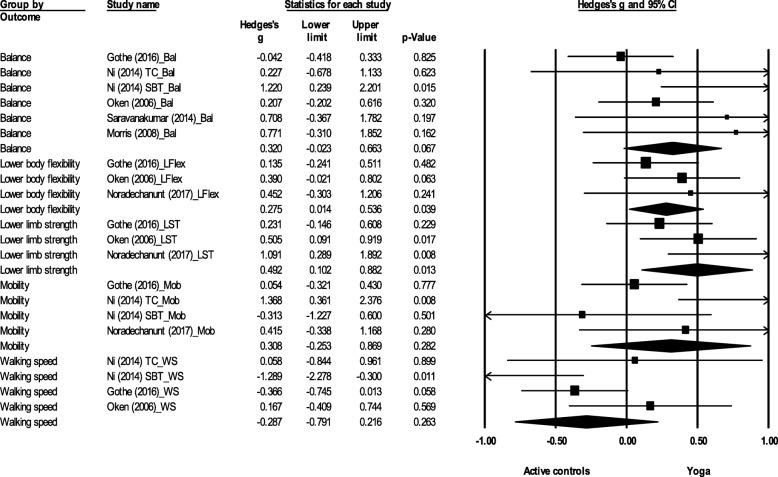
Effect sizes and 95% confidence intervals for yoga compared to active controls on physical function outcomes. Legend: Bal: balance; TC: Tai Chi; SBT: Standard balance training; LFlex: Lower body flexibility; LST: Lower limb strength; Mob: Mobility; WS: Walking speed

There was a significant effect favouring yoga for lower limb strength (ES = 0.49, 95% CI 0.10 to 0.88, *p* = 0.01) and lower body flexibility (ES = 0.28, 95% CI 0.01 to 0.54, *p* = 0.04) (Fig. 3). No significant difference between yoga and active controls was found for balance (ES = 0.32, 95% CI -0.02 to 0.66, *p* = 0.07), mobility (ES = 0.31, 95% CI -0.25 to 0.87, *p* = 0.28) or walking speed (ES = − 0.29, 95% CI -0.79 to 0.22, *p* = 0.26).

#### HRQoL

##### Yoga vs. inactive controls

**Fig. 4:**
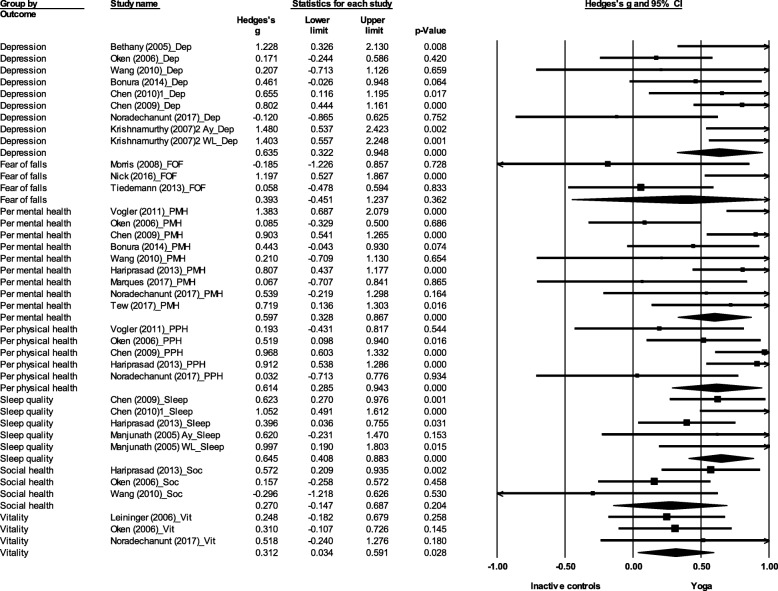
Effect sizes and 95% confidence intervals for yoga compared to inactive controls on HRQoL outcomes. Legend: Dep: Depression; FOF: Fear of falls; Per mental health/PMH: Perceived mental health; Per physical health/PPH: Perceived physical health; Sleep: Sleep quality; Soc: Social health; Vit: Vitality; Ay: Ayurveda (herbal preparation); WL: Wait-list control

There was a significant effect favouring yoga for depression (ES = 0.64, 95% CI 0.32 to 0.95, *p* < 0.001), perceived mental health (ES = 0.60, 95% CI 0.33 to 0.87, *p* < 0.001), perceived physical health (ES = 0.61, 95% CI 0.29 to 0.94, *p* < 0.001), sleep quality (ES = 0.65, 95% CI 0.41 to 0.88, *p* < 0.001), and vitality (ES = 0.31, 95% 0.03 CI to 0.59, *p* = 0.03) (Fig. 4). No significant effect was found for fear of falls (ES = 0.39, 95% CI -0.45 to 1.24, *p* = 0.36) or social health (ES = 0.27, 95% CI -0.15 to 0.69, *p* = 0.20).

##### Yoga vs. active controls

**Fig. 5:**
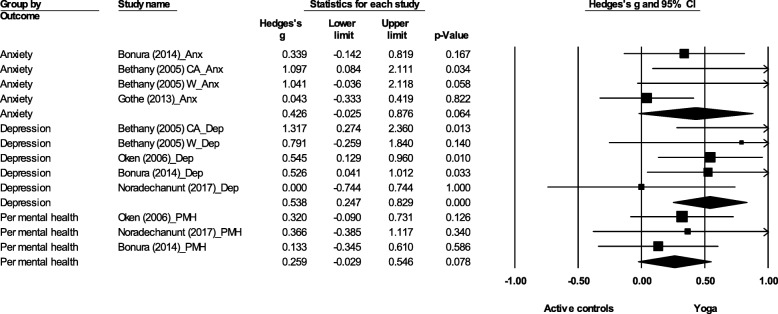
Effect sizes and 95% confidence intervals for yoga compared to active controls on HRQoL outcomes. Legend: Anx: Anxiety; CA: Chair aerobics; W: Walking programme; Dep: Depression; Per mental health/PMH: Perceived mental health

A significant effect favouring yoga was found for depression (ES = 0.54, 95% CI 0.25 to 0.83, *p* < 0.001) (Fig. 5). No significant effect was found for anxiety (ES = 0.43, 95% CI -0.03 to 0.88, *p* = 0.06) and perceived mental health (ES = 0.26, 95% CI -0.03 to 0.55, *p* = 0.08).

### Heterogeneity

Statistically significant heterogeneity was found only when comparing yoga and inactive controls (Table 4). For physical function, significant substantial heterogeneity was found for balance (I^2^ = 72.15, *p* = 0.001), and walking speed (I^2^ = 72.69, *p* = 0.003). For HRQoL outcomes, statistically significant considerable heterogeneity was found for fear of falls (I^2^ = 75.64, *p* = 0.02). Significant substantial heterogeneity was found for depression (I^2^ = 57.09, *p* = 0.02), perceived mental health (I^2^ = 54.87, p = 0.02), and perceived physical health (I^2^ = 58.55, *p* = 0.05).

Combining data from different measurement instruments could introduce heterogeneity. For example, significant heterogeneity arose in the comparison of yoga and inactive controls when balance data from one-leg-stand test, Berg balance scale, standing balance tests and POMA were combined. In contrast, when lower body flexibility was measured using a single instrument (sit-and-reach/chair sit-and-reach test) no significant heterogeneity occurred.

### Sensitivity analyses and cluster randomisation adjustment

Sensitivity analyses were conducted for four studies which had two controls, introducing one full control arm and then the other (Additional file 6). For one study [56], yoga was compared with active controls, and the sensitivity analysis affected three outcomes (balance, mobility and walking speed). For the second study [55], yoga was compared to active controls affecting two HRQoL outcomes (anxiety and depression). The third [57] and fourth study [58] compared yoga with inactive controls and the sensitivity analysis affected sleep quality [57] and depression [58]. While there were small changes in effect sizes and *p* values, none of the variables crossed the significance thresholds, and conclusions derived from the original analysis were not altered.

Meta-analysis results were not greatly altered after taking into account cluster randomization (Additional file 7). While there was a small reduction in effect sizes for some outcomes, significance was not affected.

### Risk of bias

**Fig. 6:**
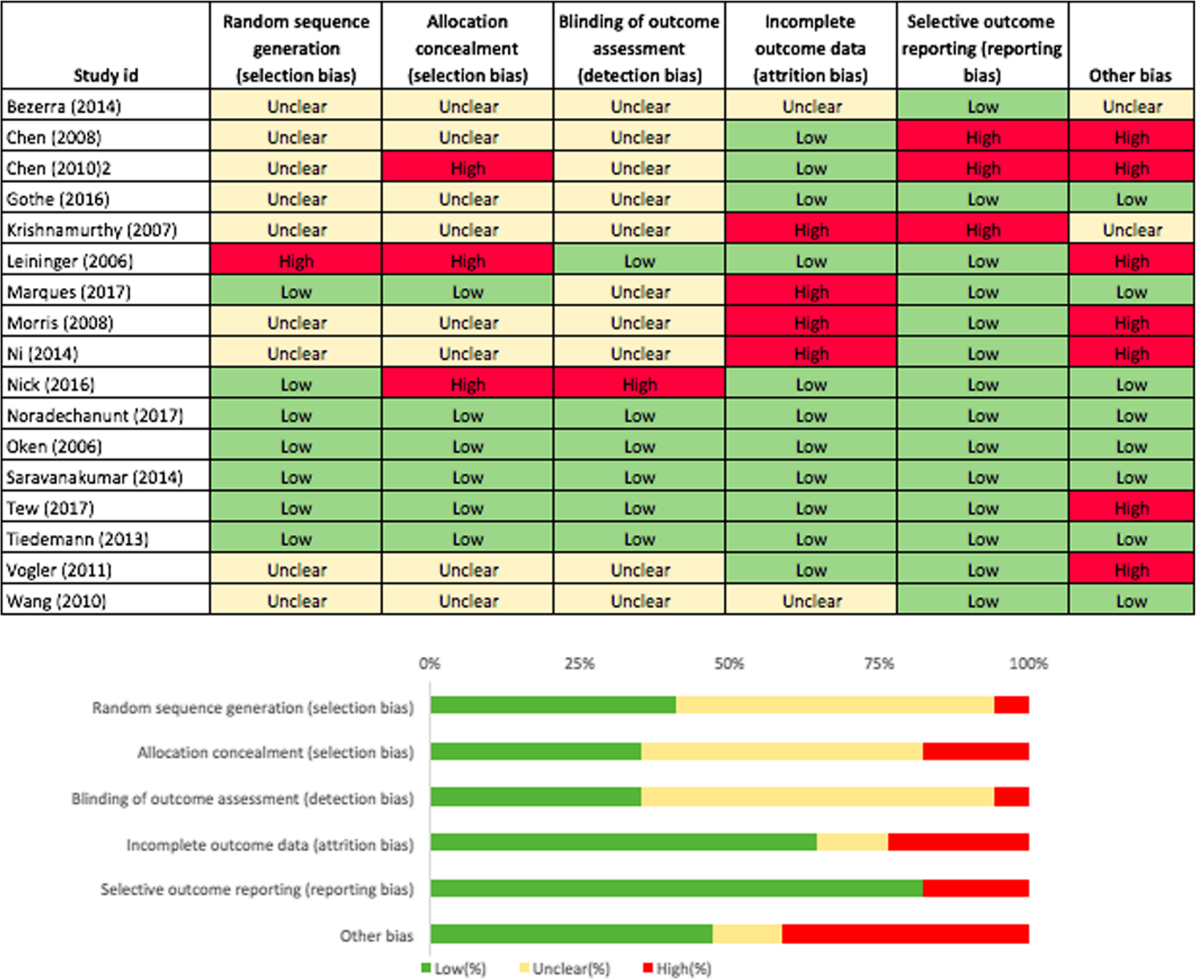
Risk of bias table and graph for physical function outcomes

**Fig. 7:**
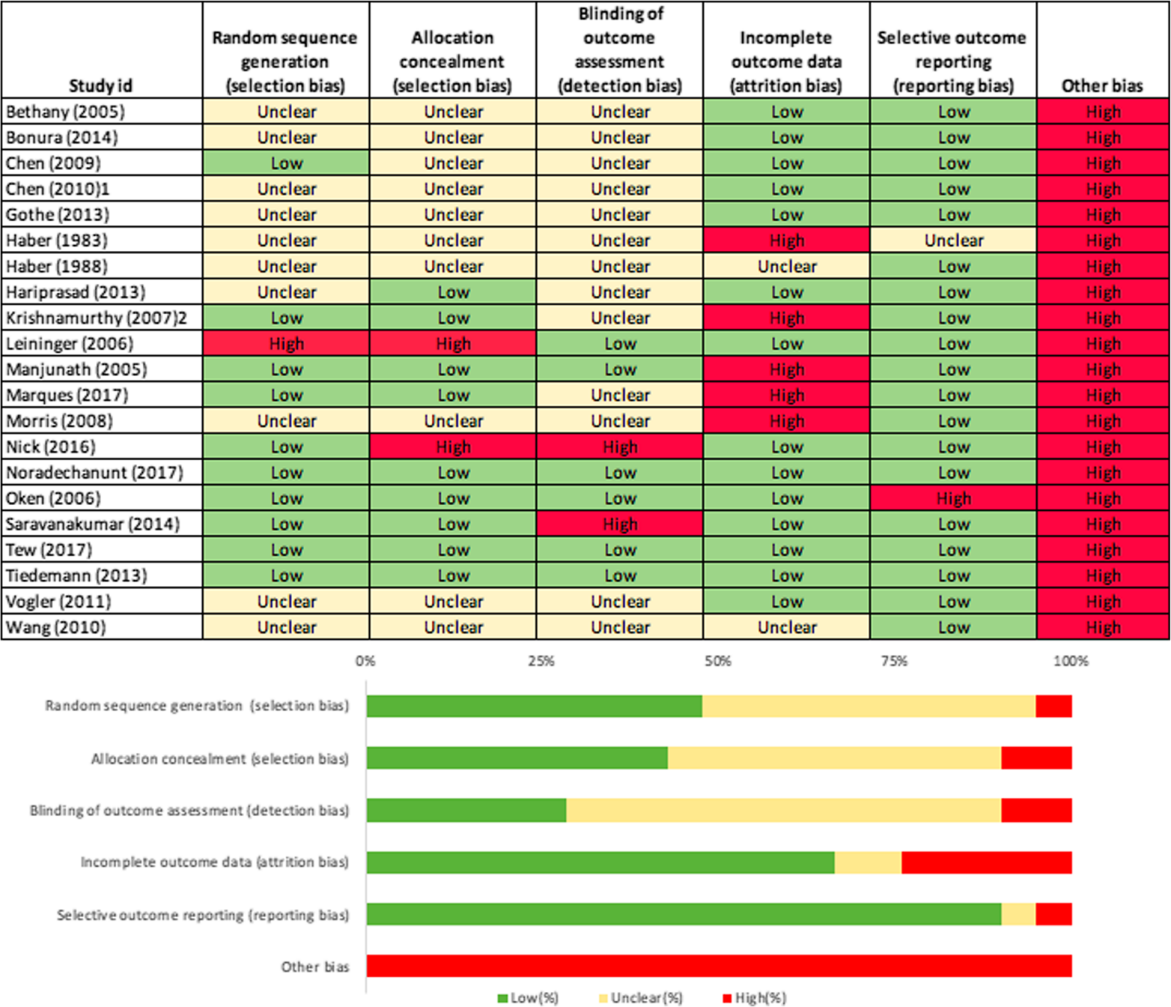
Risk of bias table and graph for HRQoL outcomes

For physical function, relatively few studies had high risk of bias (selection bias: random sequence generation (6%) and allocation concealment (18%), detection bias (6%), attrition bias (24%), reporting bias (18%) and other bias (41%)) (Fig. 6). Sample selection bias was evident for many studies and a small number were also at risk of contamination bias. Similarly, only few studies assessing HRQoL outcomes had high risk of bias (selection bias: random sequence generation (5%) and allocation concealment (10%), detection bias (10%), attrition bias (24%) and reporting bias (5%)) (Fig. 7). Other bias included response bias which emanates from the use of questionnaires and interviews, including social desirability response, acquiescence response and Hawthorne effect [48]. Since all studies assessing HRQoL used subjective self-report instruments, the risk of other bias is 100% for HRQoL outcomes. Detailed information on sources of bias is provided as supplementary material (Additional file 8).

## Discussion

### Summary of main findings

The results of this systematic review demonstrate that compared to inactive controls, it is possible for older adults to improve many aspects of their physical function and HRQoL through participating in a yoga intervention. Findings suggest that small to moderate sized beneficial effects can be achieved for balance, lower body flexibility, lower limb strength, depression, perceived mental health, perceived physical health, sleep quality, and vitality. When yoga was compared with active controls, statistically significant small to moderate effects favouring yoga were found for lower body strength, lower body flexibility and depression. Yoga was found to be as good as the activity undertaken by active controls in improving outcomes such as mobility, walking speed, balance, anxiety and perceived mental health. The yoga group was never significantly worse than the active or inactive group for any of the outcomes. With high attendance rates for class-based sessions, yoga is a feasible intervention that can be recommended to older adults as an activity that improves physical and mental wellbeing.

### Comparison to previous literature

While other systematic reviews have included or focused on studies that recruited older adults with clinical conditions, this review is the first to provide a comprehensive overview of the effects of yoga on physical function and HRQoL in an older adult population not characterised by a specific disease or condition. Outcomes such as depression, perceived mental and physical health, balance and mobility have been evaluated by other meta-analysis of RCTs in an older adult population (5), and are described in the section below.

#### Physical function

Youkhana et al. [10] conducted a systematic review to assess the effects of yoga on balance and mobility. Since the control groups in the review consisted of no intervention, waitlist control/usual care and provision of an education booklet, the study results can be contrasted with the inactive controls groups of the present study. Although the direction of the effect for balance is similar between the two reviews, the effect size in the inactive control group in this study is much higher than in the Youkhana et al. review (Table 5). The meta-analysis for balance in their review included six studies, with three studies in common with the inactive control group. The difference in effect size could be because of the extremely high effect size in one study included only in the current review, in which participants with poor balance were recruited, and saw great benefits from the yoga intervention [76]. Heterogeneity was lower and non-significant in their review for balance compared to the inactive control group in the current study. This could be attributed to more variation in the yoga types, as well as the inclusion of participants with poor balance at base-line in the current review.

**Table 5:** Comparison of effect sizes from previous reviews and the current review

Outcome	Study	Effect size in comparator review	Effect size present review	Notes
Balance	Youkhana et al. (2016)	Hedges’ g = 0.40, 95% CI 0.15 to 0.65. I^2^ = 0.00%, *p* = 0.615	Inactive controls: Hedges’ g = 0.70, 95% CI 0.19 to 1.22. I^2^ = 72.15, *p* = 0.001	Larger effect size in current review may be attributed to inclusion of a RCT [76] which recruited participants with poor balance at baseline.
Mobility	Youkhana et al. (2016)	Hedges’ g = 0.50, 95% CI 0.06 to 0.95. I^2^ = 51.8%, *p* = 0.126	Inactive controls: No meta-analysis for mobility	Mobility assessed by timed-up-and-go test in the current review (measured by only 2 studies). Mobility measured in Youkhana et al. (2016) by three studies using the timed-eight-foot-walk, sit-to-stand test and the 4-m-walk.
Perceived mental health	Tulloch et al. (2018)	Hedges’ g = 0.38, 95% CI 0.15 to 0.62. I^2^ = 56.3%, *p* = 0.009	Inactive controls: Hedges’ g = 0.6, 95% CI 0.33 to 0.87. I^2^ = 54.87, *p* = 0.02	Larger effect size in current review may be attributed to differences in inclusion criteria, differing search strategies, and search dates.
	Patel et al. (2012)	SMD = 0.66, 95% CI 0.10 to 1.22. I^2^ = 77%		Comparable effect size.
Perceived physical health	Tulloch et al. (2018)	Hedges’ g = 0.51, 95% CI 0.25 to 0.76. I^2^ = 62.9%, *p* = 0.002	Inactive controls: Hedges’ g = 0.61, 95% CI 0.29 to 0.94. I^2^ = 58.55, *p* = 0.05	Larger effect size in current study may be attributed to differences in inclusion criteria, differing search strategies, and search dates.
	Patel et al. (2012)	SMD = 0.65, 95% CI 0.02 to 1.28. I^2^ = 82%		Comparable effect size.
Depression	Patel et al. (2012)	SMD = − 0.57, 95% CI - 1.17 to 0.04. I^2^ = 80% (The negative effect size here indicates that yoga reduces depression scores to a greater extent than comparison groups)	Inactive controls: Hedges’ g = .64, 95% CI 0.32 to 0.95. I^2^ = 57.09, *p* = 0.02	Inclusion of more RCTs may have increased the power to detect differences between groups, producing significant effects favouring the yoga group in the current study.

*SMD*: Standardised Mean Difference, *CI*: Confidence Interval, *RCT*: Randomised Controlled Trial

The two reviews used different tests to assess mobility. Hence, a meta-analysis was conducted for mobility in the review by Youkhana et al. [10], but not in the current review while comparing yoga with inactive controls. The current study assessed mobility using the timed-up-and-go test (Additional file 9), which was measured only by two studies, and hence no meta-analysis was conducted. In the meta-analysis by Youkhana et al. [10], mobility was measured in three studies using the timed-eight-foot-walk, sit-to-stand test and the 4-m-walk. Two of the three studies [64, 73] were also included in the current review, with the sit-to-stand test included under strength and the 4-m-walk included under walking speed (Additional file 9).

#### HRQoL

Two reviews conducted meta-analyses to assess the effects of yoga on perceived mental and physical health in older adults [11, 13], and found a significant positive effect favouring yoga.

A smaller effect size was found for these outcomes in the Tulloch et al. review [13] compared to the current study (Table 5). The effect size in the present study for perceived physical and mental health in the inactive control group can be compared to HRQoL and mental wellbeing in the meta-analysis by Tulloch et al. correspondingly. The smaller effect size may be attributed to differences in inclusion criteria (studies which specifically recruited clinical populations were excluded in the current study), and only four of the 12 studies in the Tulloch et al. meta-analysis overlapped with the inactive control group of the present study. Some studies included in the current review [39, 65] were not captured by the Tulloch review due to differing search strategies, and search dates. The effect sizes for perceived physical and mental health in the meta-analysis by Patel et al. [11] were comparable to that of the inactive control group in the current study. Their review also assessed depression, and although a moderate effect size was found, it was not significant. The current meta-analysis for depression included more studies and may have the power to detect differences between groups. In line with the results of the current review, another systematic review published in Chinese [9] concluded that yoga significantly reduced depressive symptoms and improved quality of sleep in older adults.

### Strengths and limitations

This systematic review and meta-analysis offers a comprehensive view of the effectiveness of yoga on both physical and psychological outcomes. The method of segregating controls into active and inactive groups has not been adopted by any other systematic review for this age group, and is a significant strength of this study. The review provides novel and valuable information on the effects of yoga on some salient outcomes like strength, vitality, and social health in an older adult population. No yoga RCT has directly assessed strength in older adults using techniques like isokinetic dynamometry (gold standard) or hand-held dynamometry [79]. To our knowledge this is the first study to conduct a meta-analysis to comment on the effectiveness of yoga in improving strength albeit using a functional fitness measure as a proxy (sit-to-stand test). The sit-to-stand test is a reliable and valid indicator of lower body strength in older adults [80]. Used in conjunction with measures of flexibility, balance, mobility and walking speed, the sit-to-stand test is a fitting indicator of functional fitness and the ability to perform everyday activities in older adults [81].

This study had a broad search strategy, and criteria other than yoga and older adults were applied only at the screening stage, making it less likely to miss out studies. The review also included dissertations, which were not included in some previous reviews [11], leading to more robust results. However, the authors had difficulties in securing quantitative data for non-significant outcomes for some included studies (selective reporting bias) [53, 59], and these could not be incorporated in the meta-analysis. Consideration of this bias is critical since the primary studies test numerous outcomes, increasing the chance of type 2 errors. The inclusion of articles only published in English can be considered a limitation of the review. However, the review has captured studies from across the world including non-English speaking countries such as India, Taiwan, Brazil, and Iran. Only three studies [71, 73, 77] actually included adverse events as an outcome at the onset of the intervention. While eight studies reported on adverse events in the yoga group, it is not evident if there were no adverse events in the other studies, or if they were not reported. In one study [67] it is not clear if the injuries reported can be attributed to the yoga intervention. Ambiguous or no reporting of adverse events is a deficiency in yoga research, which future studies should address. While only a small proportion of included studies have been rated as high risk of bias, several studies have unclear risk of bias for random sequence generation, allocation concealment and blinding of outcome assessment. Future studies should ensure that randomisation and data collection procedures are reported in detail to allow for accurate assessment of bias and reliability of intervention effects.

The classification of test and instruments into broad physical function and HRQoL categories was carried out in a structured manner, referring to literature when available, to support the decisions made. However, this process can be subjective, and could be the root of differences in effect sizes between reviews (for example, sit-to-stand test was classified as assessing mobility in the Youkhana et al. review [10], but was categorised as evaluating lower limb strength in the present review).

### Implications for policy and practice

The study offers clear evidence that compared to no activity, yoga improves physical function and psychological wellbeing in older adults. It can be inferred from the meta-analysis results that yoga improves muscle strength and balance. Previous systematic reviews have highlighted the potential of yoga in improving balance in healthy adults [82], and PA policy should continue to promote yoga within muscle strength and balance guidelines to enhance and maintain health. Approximately 15% of older adults are likely to suffer from a mental health disorder [83], with depression affecting 22% of older men and 28% of older women in the UK [84]. Mental wellbeing is critical for an older adult population, and this review highlights the beneficial effects of yoga in improving perceived physical and mental health, vitality, and alleviating depressive symptoms.

The findings from this review could be used to challenge older adults’ perceptions of yoga. Older adults have the impression that yoga only improves flexibility, and the lack of an aerobic component has been cited as a barrier to yoga participation [85]. The older population and yoga teachers need to be educated on the muscle strength and balance guidelines, and also made aware of the physical function and HRQoL benefits of yoga as evidenced by this study. Information from the studies included in this review (e.g. common yoga postures and class structure) should be shared with yoga teachers. Although not directly examined in relation to effectiveness, the cross tabulation of frequency and duration of class-based sessions (Additional file 10) showed that 60 min on two days a week was the most common, which can be easily translated to practice.

Yoga is a recognised and accepted form of activity in India where it originated. In western countries, although an increasing trend in older adult participation in yoga/pilates has been observed [86–88], yoga participation rates still remain low [87, 89, 90]. This review adds to the growing evidence on the benefits of yoga, and researchers should work closely with yoga teachers, studios, fitness centres and policy makers to develop and implement strategies to encourage yoga participation among older adults, tying in with the final aim of increasing participation in muscle strength and balance activities.

### Future research

Future intervention studies should include an active control arm, so that conclusions can be drawn with respect to the effectiveness of yoga compared to different exercise programmes. Upper limb strength, hand grip strength, fall frequency, balance confidence, stress and self-efficacy are relevant and important outcomes for this population. The effects of yoga on these outcomes could not be computed through a meta-analysis due to lack of studies, and future research with robust experimental designs should focus on these outcomes. Future systematic reviews for the older adult population should aim to comment on dose-response relationships. The current review assessed the effects of yoga immediately after the intervention, and 28-weeks was the longest follow-up period. Future reviews should assess effects over a longer period, taking into account post-intervention follow-up data. Moreover, this review did not include physiological (e.g. cholesterol, indicators of immune function) and cognitive outcomes (e.g. memory and executive functions) and future reviews could aim to assess these outcomes.

There is a need to develop an appropriate framework for assessing physical function in an older adult population. Health Related Physical Fitness is defined in the American College of Sports Medicine manual as consisting of those specific components of physical fitness that have a relationship with good health, and includes cardio-respiratory fitness, body composition, muscular strength and flexibility [16]. However, it does not include mobility, walking speed, balance and frequency of falls which are important parameters of health for this population. Moreover, clear guidance is needed on the tests and instruments that assess these aspects, with details on whether they are a valid measure of the outcomes assessed. A study may have more than one instrument assessing the same outcome, and there is no standard procedure for choosing which one measure to include in the meta-analysis. This is a potential source of bias, and guidance for this process should be developed to reduce subjectivity.

## Conclusion

Results of this systematic review and meta-analysis show that yoga improves multiple physical function and HRQoL outcomes in older adults not characterised by any specific disease or condition. Compared to inactive controls, small to moderate significant effects favouring yoga were found for balance, lower body flexibility, lower limb strength, depression, perceived mental health, perceived physical health, sleep quality, and vitality. When yoga was compared with active controls, significant small to moderate effects were also found for lower body strength, lower body flexibility and depression. Yoga is a multimodal activity that improves muscle strength, balance and flexibility in older adults, and physical activity policy should continue to promote yoga as an activity that enhances physical and mental wellbeing in this population.

## Additional files

Additional file 1: Search terms for Ovid databases (MEDLINE, PsycInfo, EMBASE, AMED). Detailed list of search terms used for OVID databases are provided in this file. (PDF 37 kb)

Additional file 2: Data extraction template. This is the custom data extraction template used in the study. (XLSX 12 kb)

Additional file 3: The formula used in the study for calculating the sample size after adjusting for cluster randomisation is provided in this file. The studies included in this analysis are also listed in this document. (PDF 1175 kb)

Additional file 4: Vote count tables for physical function and HRQoL outcomes. The tables contain columns for outcome, study name, tests and instrument used, intervention and controls, and whether there were significant effects. (PDF 412 kb)

Additional file 5: (i) Data for meta-analysis- yoga compared with inactive controls for physical function outcomes. (ii) Data for meta-analysis- yoga compared with active controls for physical function outcomes. (iii) Data for meta-analysis- yoga compared with inactive controls for HRQoL outcomes. (iv) Data for meta-analysis- yoga compared with active controls for HRQoL outcomes. This excel file has four tabs which contain the data used in the main meta-analyses analyses (physical function and HRQoL outcomes for yoga vs inactive controls and yoga vs active controls) presented in this review. (XLSX 29 kb)

Additional file 6: Sensitivity analysis results. Results of the sensitivity analysis conducted are provided in this document. This includes Forest plots and homogeneity data. (PDF 307 kb)

Additional file 7: Cluster randomisation adjustment results- Forest plots and homogeneity data. Results of the cluster randomisation adjustment analysis are provided in this document. This includes Forest plots and homogeneity data. (PDF 29 kb)

Additional file 8: (i) Risk of bias details for physical function outcomes. (ii) Risk of bias details for HRQoL outcomes. This excel file has two tabs with the risk of bias details for physical function and HRQoL outcomes. (XLSX 25 kb)

Additional file 9: This table consists of the tests/instrument used to measure each outcome for all studies included in the meta-analysis (XLSX 13 kb)

Additional file 10: Frequency and duration of yoga sessions from studies included in the systematic review. This is a cross-tab of frequency and duration of yoga interventions from studies included in the meta-analysis. (XLSX 9 kb)

## Abbreviations

BMI: Body Mass Index
CI: Confidence Interval
ES: Effect Size
HRQoL: health related quality of life
MS: Muscle Strength
PA: Physical Activity
POMA: Performance Oriented Mobility Assessment
PRISMA: Preferred Reporting Items for Systematic Reviews and Meta-Analyses
RCT: Randomised Controlled Trials
ROM: Range of Motion
SMD: Standardised Mean Difference
UK: United Kingdom
US: United States
