# Supplementary material for: The effects of yoga compared to active and inactive controls on physical function and health related quality of life in older adults- systematic review and meta-analysis of randomised controlled trials
Source: Int J Behav Nutr Phys Act. 2019 Apr 5;16:33. doi: 10.1186/s12966-019-0789-2 (PMC6451238; doi:10.1186/s12966-019-0789-2)
Supplement: Supplementary file 1 — Search terms for Ovid databases (MEDLINE, PsycInfo, EMBASE, AMED). Detailed list of search terms used for OVID databases are provided in this file. (PDF 37 kb) [file 12966_2019_789_MOESM1_ESM.pdf]

Additional file 1. Search terms for Ovid databases (MEDLINE, PsycInfo, EMBASE, AMED)

1. Yoga/
2. Yoga.tw.
3. Yogic.tw.
4. Asana.tw.
5. Pranayam*.tw.
6. Dhyan*.tw.
7. 1 or 2 or 3 or 4 or 5 or 6
8. old*.tw.
9. ageing.tw.
10. aging.tw.
11. Aged/
12. Geriatrics/
13. geriatric*.tw.
14. senior*.tw.
15. elder*.tw.
16. retire*.tw.
17. pension*.tw.
18. veteran*.tw.
19. menopaus*.tw.
20. ((6? or 7? or 8? or 9?) adj2 years).tw.
21. ((6? or 7? or 8? or 9?) adj2 yr*).tw.
22. ((sixt* or sevent* or eight* or ninet*) adj2 years).tw.
23. Nursing Care/ or Nursing Homes/

24. (residential home* or residential care).tw.

25. (octogenarian* or septuagenarian* or nonagenarian* or centenarian*).tw.

26. 8 or 9 or 10 or 11 or 12 or 13 or 14 or 15 or 16 or 17 or 18 or 19 or 20 or 21 or 22 or 23 or 24 or 25

27. 7 and 26
