# Supplementary material for: The effects of yoga compared to active and inactive controls on physical function and health related quality of life in older adults- systematic review and meta-analysis of randomised controlled trials
Source: Int J Behav Nutr Phys Act. 2019 Apr 5;16:33. doi: 10.1186/s12966-019-0789-2 (PMC6451238; doi:10.1186/s12966-019-0789-2)
Supplement: Supplementary file 3 — The formula used in the study for calculating the sample size after adjusting for cluster randomisation is provided in this file. The studies included in this analysis are also listed in this document. (PDF 1175 kb) [file 12966_2019_789_MOESM3_ESM.pdf]

Additional file 3. Formula for calculation of sample size after adjusting for cluster randomisation

adjusted sample size = sample size/design effect [1]
design effect = 1 + (M-1) ICC [1]

Where
M = Average cluster size
ICC = Intracluster Correlation Coefficient

Formula for calculating ICC from an Anova table:
ICC = (MSB-MSE)/(MSB+ (K-1)MSE) [2]

Where
MSB = Between subject mean squares
MSE = Residual mean squares
K = number of observations

Data availability for studies with cluster randomized design

Studies with cluster randomisation	Data available for calculating adjusted sample size	Included in meta-analysis with sample size adjusted for cluster randomisation
Chen (2008) [3]	Yes	Yes
Chen (2009) [4]	Yes	Yes
Chen (2010)1[5]	Yes	Yes
Chen (2010) [6]	Yes	Yes
Hariprasad (2013) [7]	No	No

References

1. Higgins JP, Deeks JJ, Altman DG: **Special Topics in Statistics.** In *Cochrane Handbook for Systematic Reviews of Interventions.* Edited by P. HJ, S. G. Chichester, UK.: John Wiley & Sons, Ltd; 2008
2. Bonett DG: **Sample size requirements for estimating intraclass correlations with desired precision.** *Statistics in medicine* 2002, **21:**1331-1335.
3. Chen KM, Chen MH, Hong SM, Chao HC, Lin HS, Li CH: **Physical fitness of older adults in senior activity centers after 24-week silver yoga exercises.** *Journal of Clinical Nursing* 2008, **17:**2634-2646.
4. Chen KM, Chen MH, Chao HC, Hung HM, Lin HS, Li CH: **Sleep quality, depression state, and health status of older adults after silver yoga exercises: cluster randomized trial.** *International Journal of Nursing Studies* 2009, **46:**154-163.
5. Chen KM, Chen MH, Lin MH, Fan JT, Lin HS, Li CH: **Effects of yoga on sleep quality and depression in elders in assisted living facilities.** *Journal of Nursing Research* 2010, **18:**53-61.
6. Chen KM, Fan JT, Wang HH, Wu SJ, Li CH, Lin HS: **Silver yoga exercises improved physical fitness of transitional frail elders.** *Nursing Research* 2010, **59:**364-370.

7. Hariprasad VR, Sivakumar PT, Koparde V, Varambally S, Thirthalli J, Varghese M, Basavaraddi IV, Gangadhar BN: **Effects of yoga intervention on sleep and quality-of-life in elderly: A randomized controlled trial.** *Indian Journal of Psychiatry* 2013, **55(Suppl 3):**S364-S368.
