# Supplementary material for: The effects of yoga compared to active and inactive controls on physical function and health related quality of life in older adults- systematic review and meta-analysis of randomised controlled trials
Source: Int J Behav Nutr Phys Act. 2019 Apr 5;16:33. doi: 10.1186/s12966-019-0789-2 (PMC6451238; doi:10.1186/s12966-019-0789-2)
Supplement: Supplementary file 4 — Vote count tables for physical function and HRQoL outcomes. The tables contain columns for outcome, study name, tests and instrument used, intervention and controls, and whether there were significant effects. (PDF 412 kb) [file 12966_2019_789_MOESM4_ESM.pdf]

Additional file 4. Vote count tables for physical function and HRQoL outcomes

Physical function outcomes

Table 1. Body composition measures- yoga vs inactive controls

#	Outcome	Test/Instrument	Study	Intervention (Int) and control (Ctrl)	Effects
1	BMI	Not reported	Chen (2008)	Int1: Silver yoga<br>Int2: Short Silver yoga<br>Ctrl: Wait-list control	Significant difference favouring yoga
2	BMI	Not reported	Chen (2010)2	Int: Silver yoga<br>Ctrl: Continued daily routine	No significant effects
3	BMI	Not reported	Tew (2017)	Int: Yoga programme<br>Ctrl: Wait-list control	No significant effects
4	Body weight	Not reported	Bezerra (2014)	Int: Yoga group<br>Ctrl: Control group	No significant effects
5	Body weight	Digital body weight scale	Chen (2008)	Int1: Silver yoga<br>Int2: Short Silver yoga<br>Ctrl: Wait-list control	Significant difference favouring yoga
6	Body weight	Not reported	Chen (2010)2	Int: Silver yoga<br>Ctrl: Continued daily routine	No significant effects
7	Body weight	Not reported	Tew (2017)	Int: Yoga programme<br>Ctrl: Wait-list control	No significant effects
8	Body fat percentage	Not reported	Chen (2008)	Int1: Silver yoga<br>Int2: Short Silver yoga<br>Ctrl: Wait-list control	No significant effects
9	Body fat percentage	Body fat percentage was measured using the digital body	Chen (2010)2	Int: Silver yoga	Significant difference favouring yoga

		fat scale (model TBF521, Tanita Corporation, Kowloon, Hong Kong).		Ctrl: Continued daily routine	
10	Waist circumference	Not reported	Tew (2017)	Int: Yoga programme<br>Ctrl: Wait-list control	No significant effects

BMI- three studies measured BMI, of which one study reported a significant difference between yoga and control groups, favouring yoga
Body weight- four studies reported body weight, with only one study reporting a significant difference favouring yoga.
Body fat Percentage-Two studies measured body fat percentage, and one reported a significant difference favouring yoga.
Waist circumference- One study measured waist circumference and reported no significant effects.
On the whole, 10 results for body composition were reported with significant effects favouring yoga reported in three instances.

Table 2. Cardio-respiratory fitness- yoga vs inactive controls

#	Outcome	Test/Instrument	Study	Intervention (Int) and control (Ctrl)	Effects
1	Cardio-respiratory fitness	The 2-minute step test	Chen (2010)2	Int: Silver yoga<br>Ctrl: Continued daily routine	No significant effects
2	Cardio-respiratory fitness	6-min walk test	Noradechanunt (2017)	Int: Thai Yoga (TY)<br>Ctrl1: Tai Chi (TC)<br>Ctrl2: Telephone counselling control	Significant difference favouring yoga
3	Cardio-respiratory fitness	The 2-minute step test	Marques (2017)	Int: Chair Based Yoga<br>Ctrl: Control group	Significant difference favouring yoga

Three studies measured cardiorespiratory fitness. Significant effects favouring yoga found for two of the three studies.

Table 3. Muscle strength- yoga vs inactive controls

#	Outcome	Test/Instrument	Study	Intervention (Int) and control(Ctrl)	Effects
1	Forearm strength	Hand-grip strength	Chen (2008)	Int1: Silver yoga<br>Int2: Short Silver yoga<br>Ctrl: Wait-list control	No significant effects
2	Forearm strength	Hand-grip strength	Chen (2010)2	Int: Silver yoga<br>Ctrl: Continued daily routine	No significant effects
3	Lower limb strength	Sit-to-stand test	Oken (2006)	Int 1: Yoga<br>Ctrl1: Walking exercise<br>Ctrl2: Wait-list control	No significant effects
4	Lower limb strength	Chair-stand test	Chen (2008)	Int1: Silver yoga<br>Int2: Short Silver yoga<br>Ctrl: Wait-list control	Significant difference favouring yoga
5	Lower limb strength	Chair-stand test	Chen (2010)2	Int: Silver yoga<br>Ctrl: Continued daily routine	No significant effects
6	Lower limb strength	Chair-stand test	Noradechanunt (2017)	Int: Thai Yoga (TY)<br>Ctrl1: Tai Chi (TC)<br>Ctrl2: Telephone counselling control	Significant difference favouring yoga
7	Lower limb strength	Chair-stand test	Tew (2017)	Int: Yoga programme<br>Ctrl: Wait-list control	No significant effects
8	Lower limb strength	Sit-to-stand test	Tiedemann (2013)	Int: Yoga<br>Ctrl: Education booklet	Significant difference favouring yoga

9	Lower limb strength	Sit-to-stand test	Wang (2010)	Int: Yoga<br>Ctrl: Socialisation	No significant effects
10	Lower limb strength	Timed Floor Transfer (TFT)	Leininger (2006)	Int: Yoga<br>Ctrl: Education control	Significant difference favouring yoga
11	Muscle strength	Manual muscle testing	Vogler (2011)2	Int: Yoga<br>Ctrl: Wait-list control	Significant difference favouring yoga
12	Upper limb strength	The Arm Curl test	Chen (2010)2	Int: Silver yoga<br>Ctrl: Continued daily routine	No significant effect
13	Upper limb strength	The Arm Curl test	Noradechanunt (2017)	Int: Thai Yoga (TY)<br>Ctrl1: Tai Chi (TC)<br>Ctrl2: Telephone counselling control	Significant difference favouring yoga

13 measures of muscular strength comparing yoga and inactive controls were reported by 9 studies. In 6 studies, the yoga intervention group showed significant improvements at follow-up compared with control groups.

Eight studies measured lower limb strength, and a significant difference favouring yoga was found for four studies. The subjects in the yoga group also improved significantly compared to controls in one study measuring muscle strength. Two studies measured upper limb strength using the arm curl test, and a significant difference favouring the yoga group was found for one of the two studies. No significant differences found in the two studies measuring hand grip strength.

Table 4. Muscle strength- yoga vs active controls

#	Outcome	Test/Instrument	Study	Intervention (Int) and control (Ctrl)	Effects
1	Lower limb strength	Chair-stand test	Gothe (2016)	Int: Yoga<br>Ctrl: Stretching–Strengthening Control	No significant effects
2	Lower limb strength	Chair-stand test	Noradechanunt (2017)	Int: Thai Yoga (TY)<br>Ctrl1: Tai Chi (TC)<br>Ctrl2: Telephone counselling control	Significant difference favouring yoga
3	Lower limb strength	Sit-to-stand test	Oken (2006)	Int 1: Yoga<br>Ctrl1: Walking exercise<br>Ctrl2: Wait-list control	No significant effects
4	Upper limb strength	The Arm Curl test	Gothe (2016)	Int: Yoga<br>Ctrl: Stretching–Strengthening Control	No significant effects
5	Upper limb strength	The Arm Curl test	Noradechanunt (2017)	Int: Thai Yoga (TY)<br>Ctrl1: Tai Chi (TC)<br>Ctrl2: Telephone counselling control	No significant effects

In total, five measures of muscle strength were reported by 3 studies, and significant effects favouring yoga was reported by one study.
Three studies reported lower limb strength, and significant effects favouring yoga was reported by one study.
Two studies reported upper limb strength, with both reporting no significant interaction effects.

Table 5. **Flexibility- yoga vs inactive controls**

#	Outcome	Test/Instrument	Study	Intervention (Int) and control (Ctrl)	Effects
1	Lower body flexibility	Chair sit-and-reach test	Chen (2010)2	Int: Silver yoga<br>Ctrl: Continued daily routine	Significant difference favouring yoga
2	Lower body flexibility	Chair sit-and-reach test	Oken (2006)	Int 1: Yoga<br>Ctrl1: Walking exercise<br>Ctrl2: Wait-list control	No significant effects
3	Lower body flexibility	Sit-and-reach test	Chen (2008)	Int1: Silver yoga<br>Int2: Short Silver yoga<br>Ctrl: Wait-list control	Significant difference favouring yoga
4	Lower body flexibility	Sit-and-reach test	Wang (2010)	Int: Yoga<br>Ctrl: Socialisation	No significant effects
5	Lower body flexibility	Chair sit-and-reach test	Noradechanunt (2017)	Int: Thai Yoga (TY)<br>Ctrl1: Tai Chi (TC)<br>Ctrl2: Telephone counselling control	Significant difference favouring yoga
6	Lower body flexibility	Chair sit-and-reach test	Marques (2017)	Int: (EG) Chair Based Yoga<br>Ctrl: (CG) Control group	Significant difference favouring yoga
7	Lower body flexibility	chair sit-and-reach test	Tew (2017)	Int: Yoga programme<br>Ctrl: Wait-list control	Significant difference favouring yoga

8	Range of motion- hip abduction	Goniometer	Chen (2008)	Int1: Silver yoga<br>Int2: Short Silver yoga<br>Ctrl: Wait-list control	Significant difference favouring yoga
9	Range of motion- hip abduction	Goniometer	Chen (2010)2	Int: Silver yoga<br>Ctrl: Continued daily routine	No significant effects
10	Range of motion- hip abduction	Goniometer	Vogler (2011)	Int: Yoga<br>Ctrl: Wait-list control	Significant difference favouring yoga
11	Range of motion- hip extension	Goniometer	Vogler (2011)	Int: Yoga<br>Ctrl: Wait-list control	Significant difference favouring yoga
12	Range of motion- hip flexion	Goniometer	Chen (2008)	Int1: Silver yoga<br>Int2: Short Silver yoga<br>Ctrl: Wait-list control	Significant difference favouring yoga
13	Range of motion- hip flexion	Goniometer	Chen (2010)2	Int: Silver yoga<br>Ctrl: Continued daily routine	No significant effects
14	Range of motion- hip flexion	Goniometer	Vogler (2011)	Int: Yoga<br>Ctrl: Wait-list control	No significant effects
15	Range of motion- shoulder abduction	Goniometer	Chen (2008)	Int1: Silver yoga<br>Int2: Short Silver yoga<br>Ctrl: Wait-list control	Significant difference favouring yoga
16	Range of motion- shoulder abduction	Goniometer	Chen (2010)2	Int: Silver yoga<br>Ctrl: Continued daily routine	Significant difference favouring yoga

17	Range of motion- shoulder flexion	Goniometer	Chen (2008)	Int1: Silver yoga<br>Int2: Short Silver yoga<br>Ctrl: Wait-list control	Significant difference favouring yoga
18	Range of motion- shoulder flexion	Goniometer	Chen (2010)2	Int: Silver yoga<br>Ctrl: Continued daily routine	Significant difference favouring yoga
19	Range of motion- trunk rotation	Goniometer	Vogler (2011)	Int: Yoga<br>Ctrl: Wait-list control	Significant difference favouring yoga
20	Range of motion- upper extremity	General scan	Vogler (2011)	Int: Yoga<br>Ctrl: Wait-list control	Significant difference favouring yoga
21	Upper body flexibility	Back-scratch test	Chen (2010)2	Int: Silver yoga<br>Ctrl: Continued daily routine	Significant difference favouring yoga
22	Upper body flexibility	back-scratch test	Noradechanunt (2017)	Int: Thai Yoga (TY)<br>Ctrl1: Tai Chi (TC)<br>Ctrl2: Telephone counselling control	Significant difference favouring yoga
23	Upper body flexibility	back-scratch test	Tew (2017)	Int: Yoga programme<br>Ctrl: Wait-list control	No significant effects

A total of 23 measures of flexibility comparing yoga group to Wait-list controls were reported by 8 studies. A significant difference favouring yoga was found for 17 measures.

Out of the seven studies measuring lower body flexibility, five studies reported significant improvements in the yoga group compared to controls.

13 range of motion measures were reported by 3 studies, out of which significant improvement in the yoga group compared to controls was found for 10 measures.

Upper body flexibility using the back scratch test was measured by 3 studies, and significant difference between the groups favouring the yoga group was reported in 2 studies.

Table 6. Flexibility- yoga vs active controls

#	Outcome	Test/Instrument	Study	Intervention (Int) and control (Ctrl)	Effects
1	Lower body flexibility	Chair sit-and-reach test	Gothe (2016)	Int: Yoga<br>Ctrl: Stretching–Strengthening Control	No significant effects
2	Lower body flexibility	Chair sit-and-reach test	Oken (2006)	Int 1: Yoga<br>Ctrl1: Walking exercise<br>Ctrl2: Wait-list control	No significant effects
3	Lower body flexibility	Chair sit-and-reach test	Noradechanunt (2017)	Int: Thai Yoga (TY)<br>Ctrl1: Tai Chi (TC)<br>Ctrl2: Telephone counselling control	Significant difference favouring yoga
4	Upper body flexibility	Back-scratch test	Gothe (2016)	Int: Yoga<br>Ctrl: Stretching–Strengthening Control	No significant effects
5	Upper body flexibility	Back-scratch test	Noradechanunt (2017)	Int: Thai Yoga (TY)<br>Ctrl1: Tai Chi (TC)<br>Ctrl2: Telephone counselling control	No significant effects

3 studies reported 5 flexibility outcomes comparing yoga with active controls. Lower body flexibility was reported by three studies, with one showing a significant difference compared to active groups.

2 studies measured upper body flexibility, with no significant improvement in the yoga group compared to controls.

Table 7. Mobility and walking speed- yoga vs inactive controls

#	Outcome	Test/Instrument	Study	Intervention (Int) and control(Ctrl)	Effects
1	Mobility	Timed up and go (TUG) test	Krishnamurthy (2007)	Int: yoga<br>Ctrl: Herbal preparation	No significant effects
2	Mobility	8-foot up-and-go test	Noradechanunt (2017)	Int: Thai Yoga (TY)<br>Ctrl1: Tai Chi (TC)<br>Ctrl2: Telephone counselling control	Significant difference favouring yoga
3	Mobility	8-foot up-and-go test	Marques (2017)	Int: (EG) Chair Based Yoga<br>Ctrl: (CG) Control group	No significant effects
4	Walking speed	6-m walk test	Chen (2010)2	Int: Silver yoga<br>Ctrl: Continued daily routine	Significant difference favouring yoga
5	Walking speed	6-m walking test	Chen (2008)	Int1: Silver yoga<br>Int2: Short Silver yoga<br>Ctrl: Wait-list control	Significant difference favouring yoga
6	Walking speed	timed 4-m walk at fast pace	Tiedemann (2013)	Int: Yoga<br>Ctrl: Education booklet	Significant difference favouring yoga
7	Walking speed	timed 4-m walk at a usual pace	Tew (2017)	Int: Yoga programme<br>Ctrl: Wait-list control	No significant effects
8	Walking speed	1⁄4-mile walk	Oken (2006)	Int 1: Yoga<br>Ctrl1: Walking exercise<br>Ctrl2: Wait-list control	No significant effects

Mobility was reported by three studies, with one study reporting significant improvements in the yoga group compared to controls.

Walking speed was reported by five studies, with three reporting significant improvements in the yoga group compared to inactive controls.

Table 8. Mobility and walking speed- yoga vs active controls

#	Outcome	Test/Instrument	Study	Intervention (Int) and control(Ctrl)	Effects
1	Mobility	8-foot up-and-go test	Gothe (2016)	Int: Yoga<br>Ctrl: Stretching–Strengthening Control	No significant effects
2	Mobility	8-foot up-and-go test	Ni (2014)	Int: Yoga<br>Ctrl1: Tai Chi<br>Ctrl2: Standard balance training	No significant effects
3	Mobility	8-foot up-and-go test	Noradechanunt (2017)	Int: Thai Yoga (TY)<br>Ctrl1: Tai Chi (TC)<br>Ctrl2: Telephone counselling control	No significant effects
4	Mobility	Stairs up	Gothe (2016)	Int: Yoga<br>Ctrl: Stretching–Strengthening Control	No significant effects
5	Mobility	Stairs down	Gothe (2016)	Int: Yoga<br>Ctrl: Stretching–Strengthening Control	No significant effects
6	Walking speed	maximal walking speed	Ni (2014)	Int: Yoga<br>Ctrl1: Tai Chi<br>Ctrl2: Standard balance training	No significant effects.
7	Walking speed	Usual walking speed	Ni (2014)	Int: Yoga<br>Ctrl1: Tai Chi<br>Ctrl2: Standard balance training	No significant effects
8	Walking speed	Short Physical Performance Battery 4-meter walk	Gothe (2016)	Int: Yoga<br>Ctrl: Stretching–Strengthening Control	No significant effects
9	Walking speed	1⁄4-mile walk	Oken (2006)	Int 1: Yoga<br>Ctrl1: Walking exercise<br>Ctrl2: Wait-list control	No significant effects

Five measures of mobility were reported by three studies, and four measures of walking speed were reported by three studies. No significant differences between yoga and active controls were reported for any of the mobility or walking speed measures.

Table 9. Balance- yoga vs inactive controls

#	Outcome	Test/ Instrument	Study	Intervention (Int) and control(Ctrl)	Effects
1	Balance	One-leg-stand test	Chen (2008)	Int1: Silver yoga<br>Int2: Short Silver yoga<br>Ctrl: Wait-list control	No significant effects
2	Balance	One-leg-stand test	Chen (2010)2	Int: Silver yoga<br>Ctrl: Continued daily routine	No significant effects
3	Balance	One-leg-stand test	Oken (2006)	Int 1: Yoga<br>Ctrl1: Walking exercise<br>Ctrl2: Wait-list control	No significant effects
4	Balance	One-leg-stand test	Wang (2010)	Int: Yoga<br>Ctrl: Socialisation	No significant effects
5	Balance	One-leg-stand with eyes closed	Tiedemann (2013)	Int: Yoga<br>Ctrl: Education booklet	Significant difference favouring yoga
6	Balance	Berg Balance Scale (BBS)	Nick (2016)	Int: Yoga<br>Ctrl: Control	Significant difference favouring yoga
7	Balance	Berg Balance Scale (BBS)	Saravanakumar (2014)	Int: Yoga<br>Ctrl1: TaiChi<br>Ctrl2: Usual care	No significant effects
8	Balance	Standing balance tests	Tiedemann (2013)	Int: Yoga<br>Ctrl: Education booklet	Significant difference favouring yoga
9	Balance	Tinetti balance and gait evaluation test	Krishnamurth (2007)	Int: yoga<br>Ctrl: Herbal preparation	No significant effects

10	Balance	NeuroCom Pro Balance Master test	Morris (2008)	Int: Yoga<br>Ctrl1: Balance training exercise<br>Ctrl2: Fall risk awareness group.	No significant effects for any outcomes
11	Balance	Maximum excursion (MXE) measured by Limits of Stability (LOS)	Leininger (2006)	Int: Yoga<br>Ctrl: Education control	No significant effects for any outcomes
12	Balance	COG Sway Velocity via Unilateral stance (US) test	Leininger (2006)	Int: Yoga<br>Ctrl: Education control	No significant effects for any outcomes
13	Balance	Performance Oriented Mobility Assessment (POMA)	Morris (2008)	Int: Yoga<br>Ctrl1: Balance training exercise<br>Ctrl2: Fall risk awareness group.	No significant effects
14	Balance	Steadiness measure (SM)	Morris (2008)	Int: Yoga<br>Ctrl1: Balance training exercise<br>Ctrl2: Fall risk awareness group.	No significant effects

15	Balance	Standing balance tests	Tew (2017)	Int: Yoga programme<br>Ctrl: Wait-list control	No significant effects

15 balance measures were reported by 11 studies comparing yoga and inactive controls. Three studies reported a significant improvement for balance in the yoga group compared with the control group.

The most commonly used test for balance was the one-leg-stand test measured in five studies, with only one study reporting significant differences favouring the yoga group.

Two studies used the Berg Balance Scale (BBS), and a significant difference in mean BBS scores favouring the yoga group was found in one study.

A significant improvement in the yoga group compared with the control group was also found by Tiedemann (2013) who used standing balance tests to measure balance.

No significant differences between groups were detected for the other measures of balance.

Table 10. Balance- yoga vs active controls

#	Outcome	Test/ Instrument	Study	Intervention (Int) and control(Ctrl)	Effects
1	Balance	One-leg-stand test	Gothe (2016)	Int: Yoga<br>Ctrl: Stretching–Strengthening Control	Significant difference favouring yoga
2	Balance	One-leg-stand test	Ni (2014)	Int: Yoga<br>Ctrl1: Tai Chi<br>Ctrl2: Standard balance training	No significant effects
3	Balance	One-leg-stand test	Oken (2006)	Int 1: Yoga<br>Ctrl1: Walking exercise<br>Ctrl2: Wait-list control	No significant effects
4	Balance	Berg Balance Scale (BBS)	Saravanakumar (2014)	Int: Yoga<br>Ctrl1: TaiChi<br>Ctrl2: Usual care	No significant effects
5	Balance	Four Square Step Test	Gothe (2016)	Int: Yoga<br>Ctrl: Stretching–Strengthening Control	No significant effects
6	Balance	Functional reach (FR)	Ni (2014)	Int: Yoga<br>Ctrl1: Tai Chi<br>Ctrl2: Standard balance training	No significant effects
7	Balance	Dynamic posturography test	Ni (2014)	Int: Yoga<br>Ctrl1: Tai Chi<br>Ctrl2: Standard balance training	No significant effects for any outcomes
8	Balance	NeuroCom Pro Balance Master test	Morris (2008)	Int: Yoga<br>Ctrl1: Balance training exercise	No significant effects for any outcomes

				Ctrl2: Fall risk awareness group.	
9	Balance	Postural sway	Ni (2014)	Int: Yoga<br>Ctrl1: Tai Chi<br>Ctrl2: Standard balance training	Significant difference favouring Tai Chi for some outcomes- Eyes Closed (EC) medial- lateral displacement maximum, EC medial-lateral displacement minimum, and EC medial-lateral displacement SD
10	Balance	Performance Oriented Mobility Assessment (POMA)	Morris (2008)	Int: Yoga<br>Ctrl1: Balance training exercise<br>Ctrl2: Fall risk awareness group.	No significant effects
11	Balance	Steadiness measure (SM)	Morris (2008)	Int: Yoga<br>Ctrl1: Balance training exercise<br>Ctrl2: Fall risk awareness group.	No significant effects

11 balance measures using several instruments were reported by 5 studies. A significant effect favouring yoga was found in one study.

3 studies measured balance using the one-leg-stand, with a significant effect favouring yoga found in one study.

Ni (2014) measured postural sway. Significant effects favouring the Tai Chi group were found for Eyes Closed (EC) medial- lateral displacement maximum, EC medial-lateral displacement minimum, and EC medial-lateral displacement SD

No significant differences between groups were detected for the other measures of balance.

Table 11. Fall frequency- yoga vs inactive controls

#	Outcome	Test/Instrument	Study	Intervention (Int) and control(Ctrl)	Effects
1	Fall and instances of unsteadiness frequency	From records	Saravanakumar (2014)	Int: Yoga<br>Ctrl1: Tai Chi<br>Ctrl2: Usual care	No significant effects
2	Falls frequency	Falls self-report	Morris (2008)	Int: Yoga<br>Ctrl1: Balance training exercise<br>Ctrl2: Fall risk awareness group.	No significant effects

Falls frequency was measured by two studies. No statistically significant differences between groups detected

Table 12. Fall frequency- yoga vs active controls

#	Outcome	Test/Instrument	Study	Intervention (Int) and control(Ctrl)	Effects
1	Fall and instances of unsteadiness frequency	From records	Saravanakumar (2014)	Int: Yoga<br>Ctrl1: Tai Chi<br>Ctrl2: Usual care	No significant effects
2	Falls frequency	Falls self-report	Morris (2008)	Int: Yoga<br>Ctrl1: Balance training exercise<br>Ctrl2: Fall risk awareness group.	No significant effects

Falls frequency was measured by two studies. No statistically significant differences between groups detected.

Overall comments for physical function outcomes:

The “no significant difference” category got the most number of votes for all outcomes, when yoga was compared with active controls.

When comparing yoga groups and inactive controls, there is strong evidence to show that yoga is effective in improving flexibility where a significant difference favouring yoga was reported for 17 of 23 measures.

There is also evidence to show that yoga improves walking speed compared to inactive controls with three of five studies showing a significant improvement in the yoga group compared to controls.

Health related quality of life (HRQoL) outcomes

Table 1. Anxiety- yoga vs inactive controls

#	Outcome	Test/Instrument	Study	Intervention (Int) and controls (Ctrl)	Effects
1	Anxiety	State Anxiety Inventory	Bonura (2014)	Int: Chair yoga<br>Ctrl: Chair Exercise<br>Ctrl: Wait-list control group	Significant difference favouring yoga
2	Anxiety	State Anxiety Inventory	Bethany (2005)	Int: Chair yoga<br>Ctrl: Chair aerobics<br>Ctrl: Walking program<br>Ctrl: Game playing group	No significant effects

Two studies reported on anxiety, and a significant effect favouring yoga was found in one study

Table 2. Anxiety- yoga vs active controls

#	Outcome	Test/Instrument	Study	Intervention (Int) and control (Ctrl)	Effects
1	Anxiety	State Anxiety Inventory	Bonura (2014)	Int: Chair yoga<br>Ctrl: Chair Exercise<br>Ctrl: Wait-list control group	Significant difference favouring yoga
2	Anxiety	State scale of State-Trait Anxiety Inventory	Bethany (2005)	Int: Chair yoga<br>Ctrl: Chair aerobics<br>Ctrl: Walking program<br>Ctrl: Game playing group	No significant effects
3	Anxiety	State scale of State-Trait Anxiety Inventory	Gothe (2013)	Int: Yoga Group<br>Ctrl: Stretching Control Group	No significant effects
4	Anxiety	Trait scale of State-Trait Anxiety Inventory	Gothe (2013)	Int: Yoga Group<br>Ctrl: Stretching Control Group	No significant effects

Four measures from three studies reported on anxiety. One result with a significant effect favouring yoga was reported.

Table 3. Depression- yoga vs inactive controls

#	Outcome	Test/Instrument	Study	Intervention (Int) and control (Ctrl)	Effects
1	Depression	Beck Depression Inventory – II	Bethany (2005)	Int: Chair yoga<br>Ctrl: Chair aerobics<br>Ctrl: Walking program<br>Ctrl: Game playing group	No significant effects
2	Depression	Epidemiological Studies Depression Scale (CESD-10)	Oken (2006)	Int 1: Yoga<br>Ctrl1: Walking exercise<br>Ctrl2: Wait-list control	No significant effects
3	Depression	Epidemiological Studies Depression Scale (CES-D)	Wang (2010)	Int: Yoga<br>Ctrl: Socialisation	No significant effects
4	Depression	Geriatric Depression Scale	Bonura (2014)	Int: Chair yoga<br>Ctrl: Chair Exercise<br>Ctrl: Wait-list control group	Significant difference favouring yoga
5	Depression	Taiwanese Depression Questionnaire	Chen (2010)1	Int: yoga intervention group<br>Ctrl: Wait-list control group	No significant effects

6	Depression	Taiwanese Depression Questionnaire	Chen (2009)	Int: silver yoga experimental group<br>Ctrl: Wait-list control group	Significant difference favouring yoga
7	Depression	Epidemiological Studies Depression Scale (CES-D)	Noradechanunt (2017)	Int: Thai Yoga (TY)<br>Ctrl1: Tai Chi (TC)<br>Ctrl2: Telephone counselling control	No significant effects
8	Depression	Short version of the Geriatric Depression Scale	Krishnamurthy (2007) 2	Int: Yoga training<br>Ctrl: Ayurveda<br>Ctrl: Wait-list control	Significant difference favouring yoga

Eight studies reported depression. A significant improvement in the yoga group compared to controls was found for three studies.

Table 4. Depression- yoga vs active controls

#	Outcome	Test/Instrument	Study	Intervention (Int) and control (Ctrl)	Effects
1	Depression	Beck Depression Inventory – II	Bethany (2005)	Int: Chair yoga<br>Ctrl: Chair aerobics<br>Ctrl: Walking program<br>Ctrl: Game playing group	No significant effects
2	Depression	Epidemiological Studies Depression Scale (CES-D)	Oken (2006)	Int 1: Yoga<br>Ctrl1: Walking exercise<br>Ctrl2: Wait-list control	No significant effects
3	Depression	Geriatric Depression Scale	Bonura (2014)	Int: Chair yoga<br>Ctrl: Chair Exercise<br>Ctrl: Wait-list control group	Significant difference favouring yoga
4	Depression	Epidemiological Studies Depression Scale (CES-D)	Noradechanunt (2017)	Int: Thai Yoga (TY)<br>Ctrl1: Tai Chi (TC)<br>Ctrl2: Telephone counselling control	No significant effects

Four studies measured depression, and a significant effect favouring yoga was reported for 1 study.

Table 5. Perceived physical health - yoga vs inactive controls

#	Outcome	Test/Instrument	Study	Intervention (Int) and control (Ctrl)	Effects
1	Perceived physical health	Physical Component Summary of the SF-12v2	Vogler (2011)	Int: Yoga<br>Ctrl: Wait-list control	No significant effects
2	Perceived physical health	Physical Health Composite Summary score of the Short Form-36	Oken (2006)	Int 1: Yoga<br>Ctrl1: Walking exercise<br>Ctrl2: Wait-list control	No significant effects
3	Perceived physical health	Physical health component SF-12 Healthy Survey, Chinese version	Chen (2009)	Int: silver yoga experimental group<br>Ctrl: Wait-list control group	Significant difference favouring yoga
4	Perceived physical health	WHOQOL-BREF-Physical health domain	Hariprasad, (2013)	Int: Yoga<br>Ctrl: Wait-list control	Significant difference favouring yoga
5	Perceived physical health	Physical Health Composite Summary score of the Short Form-36	Noradechanunt (2017)	Int: Thai Yoga (TY)<br>Ctrl1: Tai Chi (TC)<br>Ctrl2: Telephone counselling control	No significant effects

6	Perceived physical health	Self-Assessed Health Status	Haber (1983)	Int: Yoga<br>Ctrl: Control group	Significant effects favouring yoga in one centre. Inconclusive overall effects.

Six studies measured perceived physical health, and two reported a significant difference favouring yoga. In Haber (1983), only one centre showed significant effect favouring yoga, and overall effects were not presented.

Table 6. Perceived physical health - yoga vs active controls

#	Outcome	Test/Instrument	Study	Intervention (Int) and control (Ctrl)	Effects
1	Perceived physical health	Physical Health Composite Summary score of the Short Form-36	Oken (2006)	Int 1: Yoga<br>Ctrl1: Walking exercise<br>Ctrl2: Wait-list control	No significant effects
2	Perceived physical health	Physical Health Composite Summary score of the Short Form-36	Noradechanunt (2017)	Int: Thai Yoga (TY)<br>Ctrl1: Tai Chi (TC)<br>Ctrl2: Telephone counselling control	No significant effects

Two studies measured perceived physical health, and none reported a significant difference favouring yoga.

Table 7. Perceived mental health - yoga vs inactive controls

#	Outcome	Test/Instrument	Study	Intervention (Int) and control (Ctrl)	Effects
1	Perceived mental health	Mental Component Summary of the SF-12v2	Vogler (2011)	Int: Yoga<br>Ctrl: Wait-list control	No significant effects
2	Perceived mental health	Mental Health Composite Summary score of the Short Form-36	Oken (2006)	Int 1: Yoga<br>Ctrl1: Walking exercise<br>Ctrl2: Wait-list control	No significant effects
3	Perceived mental health	Mental health component of SF-12 Healthy Survey, Chinese version	Chen et (2009)	Int: silver yoga experimental group<br>Ctrl: Wait-list control group	Significant difference favouring yoga
4	Perceived mental health	Lawton's PGC Morale Scale	Bonura (2014)	Int: Chair yoga<br>Ctrl: Chair Exercise<br>Ctrl: Wait-list control group	Significant difference favouring yoga
5	Perceived mental health	Philadelphia Geriatric Center Morale Scale- Revised (PGMS)	Wang (2010)	Int: Yoga<br>Ctrl: Socialisation	No significant effects
6	Perceived mental health	WHOQOL-BREF- mental health domain	Hariprasad, (2013)	Int: Yoga<br>Ctrl: Wait-list control	Significant difference favouring yoga

7	Perceived mental health	WHO-5	Marques (2017)	Int: (EG) Chair Based Yoga<br>Ctrl: (CG) Control group	No significant effects
8	Perceived mental health	Mental Health Composite Summary score of the Short Form-36	Noradechanunt (2017)	Int: Thai Yoga (TY)<br>Ctrl1: Tai Chi (TC)<br>Ctrl2: Telephone counselling control	No significant effects
9	Perceived mental health	WEMWBS	Tew (2017)	Int: Yoga programme<br>Ctrl: Wait-list control	Significant difference favouring yoga
10	Perceived mental health	Psychological Well-Being	Haber (1983)	Int: Yoga<br>Ctrl: Control group	Significant effects favouring yoga in one centre. Overall effects of yoga not presented.

Ten studies reported perceived mental health, of which significant effects favouring yoga was found in four studies. In Haber (1983), only one centre showed significant effect favouring yoga, and overall effects were not presented.

Table 8. Perceived mental health - yoga vs active controls

#	Outcome	Test/Instrument	Study	Intervention (Int) and control (Ctrl)	Effects
1	Perceived mental health	Mental Health Composite Summary score of the Short Form-36	Oken (2006)	Int 1: Yoga<br>Ctrl1: Walking exercise<br>Ctrl2: Wait-list control	No significant effects

2	Perceived mental health	Mental Health Composite Summary score of the Short Form-36	Noradechanunt (2017)	Int: Thai Yoga (TY)<br>Ctrl1: Tai Chi (TC)<br>Ctrl2: Telephone counselling control	No significant effects
3	Perceived mental health	Lawton's PGC Morale Scale	Bonura (2014)	Int: Chair yoga<br>Ctrl: Chair Exercise<br>Ctrl: Wait-list control group	Significant difference favouring yoga

Three studies measured perceived mental health with one reporting significant effects favouring yoga.

Table 9. Vitality- yoga vs inactive controls

#	Outcome	Test/Instrument	Study	Intervention (Int) and control (Ctrl)	Effects
1	Vitality	Vitality scale of SF-36	Leininger (2006)	Int: Yoga<br>Ctrl: Education control	No significant effects
2	Vitality	Vitality scale of SF-36	Oken (2006)	Int 1: Yoga<br>Ctrl1: Walking exercise<br>Ctrl2: Wait-list control	No significant effects
3	Vitality	Vitality scale of SF-36	Noradechanunt (2017)	Int: Thai Yoga (TY)<br>Ctrl1: Tai Chi (TC)<br>Ctrl2: Telephone counselling control	Significant difference favouring yoga

Three studies measured vitality, with one reporting significant effects favouring yoga.

Table 10. Vitality- yoga vs active controls

#	Outcome	Test/Instrument	Study	Intervention (Int) and control (Ctrl)	Effects
1	Vitality	Vitality scale of SF-36	Oken (2006)	Int 1: Yoga<br>Ctrl1: Walking exercise<br>Ctrl2: Wait-list control	No significant effects
2	Vitality	Vitality scale of SF-36	Noradechanunt (2017)	Int: Thai Yoga (TY)<br>Ctrl1: Tai Chi (TC)<br>Ctrl2: Telephone counselling control	Significant difference favouring yoga

Two studies measured vitality, of which one reported significant effects favouring yoga.

Table 11. Quality of life- yoga vs inactive controls

#	Outcome	Test/Instrument	Study	Intervention (Int) and control (Ctrl)	Effects
1	Quality of life	dementia quality of life (DQOL) questionnaire	Saravanakumar (2014)	Int: Yoga<br>Ctrl1: TaiChi<br>Ctrl2: Usual care	No significant effects
2	Quality of life	EQ-5D utility index	Tew (2017)	Int: Yoga programme<br>Ctrl: Wait-list control	Significant difference favouring yoga
3	Quality of life	EQ-VAS	Tew (2017)	Int: Yoga programme<br>Ctrl: Wait-list control	Significant difference favouring yoga

Three measures of quality of life were reported by two studies. Two results with significant effects favouring yoga were reported.

Table 12. Social health- yoga vs inactive controls

#	Outcome	Test/Instrument	Study	Intervention (Int) and control (Ctrl)	Effects
1	Social Health	WHOQOL-BREF- social relationships domain	Hariprasad, (2013)	Int: Yoga<br>Ctrl: Wait-list control	Significant difference favouring yoga
2	Social Health	Social functioning from the Short Form-36	Oken (2006)	Int 1: Yoga<br>Ctrl1: Walking exercise	No significant effects

				Ctrl2: Wait-list control	
3	Social Health	UCLA Loneliness Scale	Wang (2010)	Int: Yoga<br>Ctrl: Socialisation	No significant effects
4	Social Health	Self-reported frequency of conversation and perceived closeness to others in the nursing home	Haber (1988)	Int: Yoga<br>Ctrl: Control group	No significant effects

Four studies measured social health, of which one reported significant effects favouring yoga.

Table 13. Sleep quality- yoga vs inactive controls

#	Outcome	Test/Instrument	Study	Intervention (Int) and control (Ctrl)	Effects
1	Sleep quality	Pittsburgh Sleep Quality Index (PSQI)<br>PSQI total score	Chen (2009)	Int: silver yoga experimental group<br>Ctrl: Wait-list control group	Significant difference favouring yoga
2	Sleep quality	Pittsburgh Sleep Quality Index (PSQI)<br>PSQI total score	Chen (2010)1	Int: yoga intervention group<br>Ctrl: Wait-list control group	Significant difference favouring yoga

3	Sleep quality	Pittsburgh Sleep Quality Index (PSQI)<br>PSQI total score	Hariprasad (2013)	Int: Yoga<br>Ctrl: Wait-list control	Significant difference favouring yoga
4	Sleep quality	sleep rating questionnaire: 5 questions	Manjunath (2005)	Int: Yoga training<br>Ctrl: Ayurveda<br>Ctrl: Wait-list control	No significant effects

Four studies measured sleep quality, and three reported significant effects favouring yoga.

Table 14. Stress- yoga vs inactive control

#	Outcome	Test/Instrument	Study	Intervention (Int) and control (Ctrl)	Effects
1	Stress Frequency	Hassles Scale	Bethany (2005)	Int: Chair yoga<br>Ctrl: Chair aerobics<br>Ctrl: Walking program<br>Ctrl: Game playing group	Significant difference favouring yoga
2	Stress Severity	Hassles Scale	Bethany (2005)	Int: Chair yoga<br>Ctrl: Chair aerobics<br>Ctrl: Walking program<br>Ctrl: Game playing group	No significant effects
3	Stress	Perceived Stress Scale 14-item Perceived Stress Scale	Marques (2017)	Int: (EG) Chair Based Yoga<br>Ctrl: (CG) Control group	No significant effects
4	Stress	Salivary Cortisol (pre and post stressor)	Marques (2017)	Int: (EG) Chair Based Yoga<br>Ctrl: (CG) Control group	No significant effects

Four results for stress reported by two studies. Significant effects favouring yoga reported for only one result.

Table 15. Stress- yoga vs active controls

#	Outcome	Test/Instrument	Study	Intervention (Int) and control (Ctrl)	Effects
1	Stress	Perceived Stress Scale 14-item Perceived Stress Scale	Gothe (2013)	Int: Yoga Group<br>Ctrl: Stretching Control Group	No significant effects
2	Stress	Salivary Cortisol (pre and post stressor)	Gothe (2013)	Int: Yoga Group<br>Ctrl: Stretching Control Group	No significant effects
3	Stress Frequency	Hassles Scale	Bethany (2005)	Int: Chair yoga<br>Ctrl: Chair aerobics<br>Ctrl: Walking program<br>Ctrl: Game playing group	Significant difference favouring yoga
4	Stress Severity	Hassles Scale	Bethany (2005)	Int: Chair yoga<br>Ctrl: Chair aerobics<br>Ctrl: Walking program<br>Ctrl: Game playing group	No significant effects

Four results for stress reported by two studies. Significant effects favouring yoga reported for only one result.

Table 16. Fear of falls- yoga vs inactive controls

#	Outcome	Test/Instrument	Study	Intervention (Int) and control (Ctrl)	Effects
1	Fear of falls	Fear Efficacy Scale	Morris (2008)	Int: Yoga<br>Ctrl1: Balance training exercise<br>Ctrl2: Fall risk awareness group	No significant effects
2	Fear of falls	Modified Falls Efficacy Scale (MFES)	Nick (2016)	Int: Yoga<br>Ctrl: Control	Significant difference favouring yoga
3	Fear of falls	Short Falls Efficacy Scale-International (Short FES-I)	Tiedemann (2013)	Int: Yoga<br>Ctrl: Education booklet	No significant effects

Three studies measured fear of falls, of which one reported significant effects favouring yoga.

Table 17. Balance confidence- yoga vs inactive controls

#	Outcome	Test/Instrument	Study	Intervention (Int) and control (Ctrl)	Effects
1	Balance Confidence	Activities-Specific Balance Confidence (ABC) Scale	Leininger (2006)	Int: Yoga<br>Ctrl: Education control	No significant effects
2	Balance Confidence	Activities-Specific Balance Confidence (ABC) Scale	Morris (2008)	Int: Yoga<br>Ctrl1: Balance training exercise<br>Ctrl2: Fall risk awareness group.	No significant effects

Two studies measured balance confidence, and none reported significant effects favouring yoga.

Table 18. General Health and Well-Being- yoga vs inactive controls

#	Outcome	Test/Instrument	Study	Intervention (Int) and control (Ctrl)	Effects
1	General Health and Well-Being	SF-12v2 (1st Q)	Vogler (2011)	Int: Yoga<br>Ctrl: Wait-list control	No significant effects
2	General Health and Well-Being	General health from Short Form-36	Oken (2006)	Int 1: Yoga<br>Ctrl1: Walking exercise<br>Ctrl2: Wait-list control	No significant effect

Two studies measured balance confidence, and none reported significant effects favouring yoga.

Overall comments for HRQoL outcomes:

As with physical function, when comparing yoga and active controls, the “significant effect favouring yoga” category was not found to be the “winner” with most number of votes for any of the outcomes.

Comparing yoga and inactive controls, the yoga group got the most votes for quality of life (two of three studies reported significant effects favouring yoga), and sleep quality (three of four studies reported significant effects favouring yoga).
