# Supplementary material for: The effects of yoga compared to active and inactive controls on physical function and health related quality of life in older adults- systematic review and meta-analysis of randomised controlled trials
Source: Int J Behav Nutr Phys Act. 2019 Apr 5;16:33. doi: 10.1186/s12966-019-0789-2 (PMC6451238; doi:10.1186/s12966-019-0789-2)
Supplement: Supplementary file 6 — Sensitivity analysis results. Results of the sensitivity analysis conducted are provided in this document. This includes Forest plots and homogeneity data. (PDF 307 kb) [file 12966_2019_789_MOESM6_ESM.pdf]

Additional file 6. Sensitivity analysis results

Sensitivity analysis conducted for 1. Bethany (2005) [1], 2. Ni (2014) [2], 3. Manjunath (2005) [3], 4. Krishnamurthy (2007)2 [4]

Four studies [1-4] had one yoga intervention group and two control groups. In these cases, the result was included twice in the meta-analysis with half the number of participants for the yoga group each time. Following this, two sensitivity analyses were also conducted: (i) comparing the full yoga intervention arm and the first control group, and (ii) comparing the full yoga intervention arm and the second control group.

The results for the sensitivity analysis for each study are given below:

1. Bethany (2005) [1]

Sensitivity analysis 1: Meta-analysis results (HRQoL- yoga vs. active controls) choosing yoga group and chair aerobics for Bethany (2005)

Group by Outcome	Study name	Statistics for each study: Hedges's g	Lower limit	Upper limit	p-Value
Anxiety	Bonura (2014)_Anx	0.339	-0.142	0.819	0.167
Anxiety	Bethany (2005) CA_Anx	1.067	0.203	1.930	0.015
Anxiety	Gothe (2013)_Anx	0.043	-0.333	0.419	0.822
Anxiety		0.356	-0.118	0.830	0.141
Depression	Bethany (2005) CA_Dep	1.313	0.420	2.206	0.004
Depression	Oken (2006)_Dep	0.545	0.129	0.960	0.010
Depression	Bonura (2014)_Dep	0.526	0.041	1.012	0.033
Depression	Noradechanunt (2017)_Dep	0.000	-0.744	0.744	1.000
Depression		0.546	0.170	0.922	0.004
Per mental health	Oken (2006)_PMH	0.320	-0.090	0.731	0.126
Per mental health	Noradechanunt (2017)_PMH	0.366	-0.385	1.117	0.340
Per mental health	Bonura (2014)_PMH	0.133	-0.345	0.610	0.586
Per mental health		0.259	-0.029	0.546	0.078

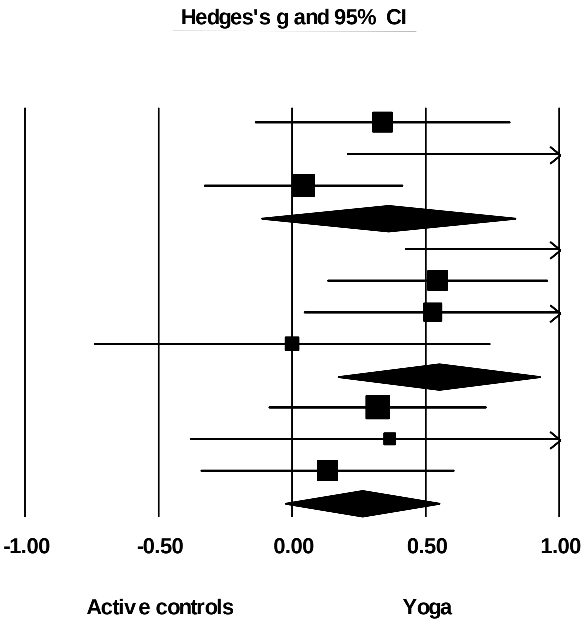

CA: Chair aerobics; Anx: Anxiety; Dep: Depression; Per mental health/PMH: Perceived mental health

Heterogeneity data

Outcome	I square	P value
Anxiety	57.74	0.09
Depression	38.82	0.18
Perceived mental health	0	0.81

Sensitivity analysis 2: Meta-analysis results (HRQoL- yoga vs. active controls) choosing yoga group and walking programme for Bethany (2005)

Group by Outcome	Study name	Hedges's g	Lower limit	Upper limit	p-Value
		Statistics for each study			
Anxiety	Bonura (2014)_Anx	0.339	-0.142	0.819	0.167
Anxiety	Bethany (2005) W_Anx	1.030	0.151	1.910	0.022
Anxiety	Gothe (2013)_Anx	0.043	-0.333	0.419	0.822
Anxiety		0.338	-0.116	0.791	0.145
Depression	Bethany (2005) W_Dep	0.884	0.019	1.748	0.045
Depression	Oken (2006)_Dep	0.545	0.129	0.960	0.010
Depression	Bonura (2014)_Dep	0.526	0.041	1.012	0.033
Depression	Noradechanunt (2017)_Dep	0.000	-0.744	0.744	1.000
Depression		0.499	0.223	0.774	0.000
Per mental health	Oken (2006)_PMH	0.320	-0.090	0.731	0.126
Per mental health	Noradechanunt (2017)_PMH	0.366	-0.385	1.117	0.340
Per mental health	Bonura (2014)_PMH	0.133	-0.345	0.610	0.586
Per mental health		0.259	-0.029	0.546	0.078

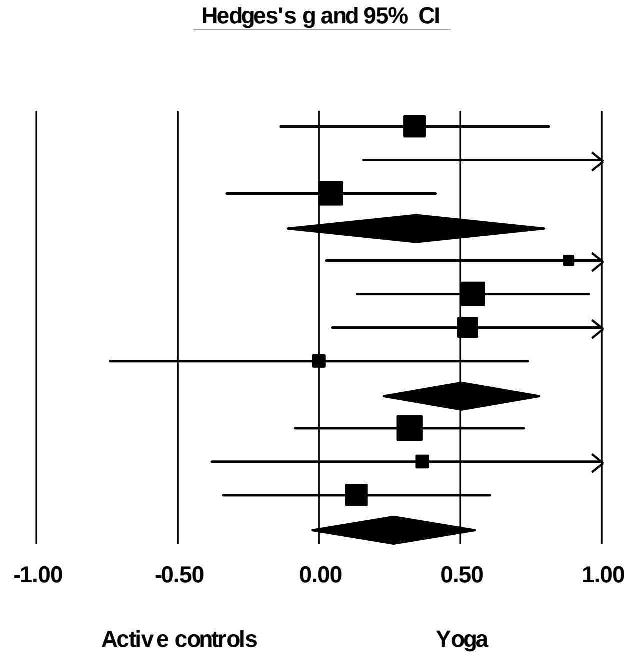

W: Walking programme; Anx: Anxiety; Dep: Depression; Per mental health/PMH: Perceived mental health

Heterogeneity data

Outcome	I square	P value
Anxiety	53.73	0.15
Depression	0	0.47
Perceived mental health	0	0.81

2. Ni (2014) [2]

Sensitivity analysis 1: Meta-analysis results (Physical function- yoga vs. active controls) choosing yoga group and standard balance training (SBT) for Ni (2014)

Group by Outcome	Study name	Hedges's g	Lower limit	Upper limit	p-Value
		Statistics for each study			
Balance	Gothe (2016)_Bal	-0.042	-0.418	0.333	0.825
Balance	Ni (2014) SBT_Bal	1.216	0.428	2.004	0.002
Balance	Oken (2006)_Bal	0.207	-0.202	0.616	0.320
Balance	Saravanakumar (2014)_Bal	0.708	-0.367	1.782	0.197
Balance	Morris (2008)_Bal	0.771	-0.310	1.852	0.162
Balance		0.434	-0.013	0.881	0.057
Lower body flexibility	Gothe (2016)_Lflex	0.135	-0.241	0.511	0.482
Lower body flexibility	Oken (2006)_LFlex	0.390	-0.021	0.802	0.063
Lower body flexibility	Noradechanunt (2017)_Lflex	0.452	-0.303	1.206	0.241
Lower body flexibility		0.275	0.014	0.536	0.039
Lower limb strength	Gothe (2016)_LST	0.231	-0.146	0.608	0.229
Lower limb strength	Oken (2006)_LST	0.505	0.091	0.919	0.017
Lower limb strength	Noradechanunt (2017)_LST	1.091	0.289	1.892	0.008
Lower limb strength		0.492	0.102	0.882	0.013
Mobility	Gothe (2016)_Mob	0.054	-0.321	0.430	0.777
Mobility	Ni (2014) SBT_Mob	-0.312	-1.038	0.413	0.399
Mobility	Noradechanunt (2017)_Mob	0.415	-0.338	1.168	0.280
Mobility		0.049	-0.256	0.354	0.754
Walking speed	Ni (2014) SBT_WS	-1.287	-2.082	-0.491	0.002
Walking speed	Gothe (2016)_WS	-0.366	-0.745	0.013	0.058
Walking speed	Oken (2006)_WS	0.167	-0.409	0.744	0.569
Walking speed		-0.439	-1.110	0.232	0.200

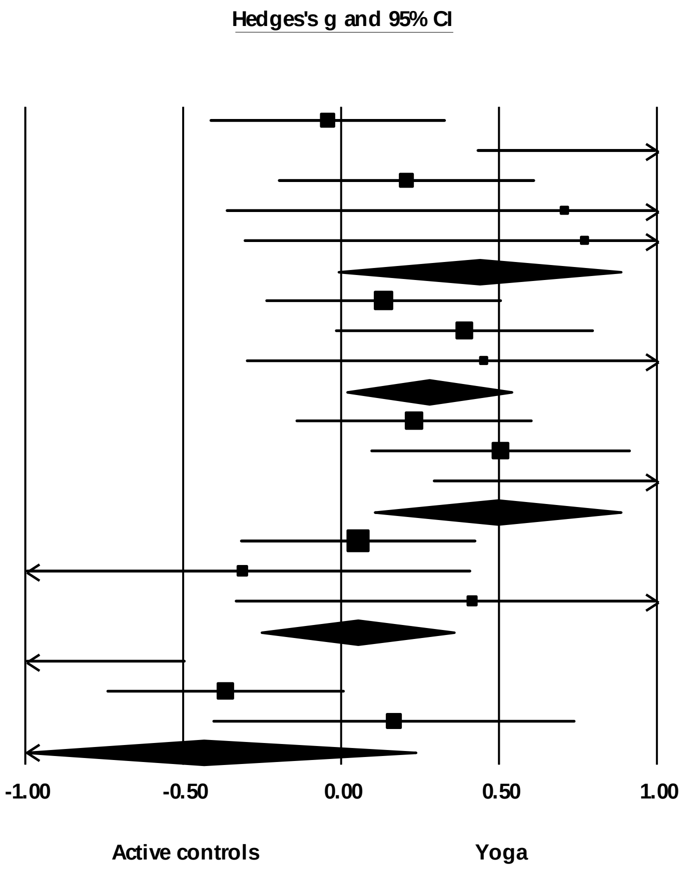

SBT: Standard balance training; Lflex: Lower body flexibility; LST: Lower limb strength; Mob: Mobility; WS: Walking speed

Heterogeneity data

Outcome	I square	P value
Balance	58.89	0.05
Lower body flexibility	0	0.59
Lower limb strength	47.44	0.15
Mobility	0	0.35
Walking speed	76.25	0.02

Sensitivity analysis 2: Meta-analysis results (Physical function- yoga vs. active controls) choosing yoga group and Tai Chi (TC) group for Ni (2014)

Group by Outcome	Study name	Hedges's g	Lower limit	Upper limit	p-Value
		Statistics for each study			
Balance	Gothe (2016)_Bal	-0.042	-0.418	0.333	0.825
Balance	Ni (2014) TC_Bal	0.233	-0.545	1.012	0.556
Balance	Oken (2006)_Bal	0.207	-0.202	0.616	0.320
Balance	Saravanakumar (2014)_Bal	0.708	-0.367	1.782	0.197
Balance	Morris (2008)_Bal	0.771	-0.310	1.852	0.162
Balance		0.158	-0.089	0.405	0.209
Lower body flexibility	Gothe (2016)_Lflex	0.135	-0.241	0.511	0.482
Lower body flexibility	Oken (2006)_LFlex	0.390	-0.021	0.802	0.063
Lower body flexibility	Noradechanunt (2017)_Lflex	0.452	-0.303	1.206	0.241
Lower body flexibility		0.275	0.014	0.536	0.039
Lower limb strength	Gothe (2016)_LST	0.231	-0.146	0.608	0.229
Lower limb strength	Oken (2006)_LST	0.505	0.091	0.919	0.017
Lower limb strength	Noradechanunt (2017)_LST	1.091	0.289	1.892	0.008
Lower limb strength		0.492	0.102	0.882	0.013
Mobility	Gothe (2016)_Mob	0.054	-0.321	0.430	0.777
Mobility	Ni (2014) TC_Mob	1.409	0.537	2.280	0.002
Mobility	Noradechanunt (2017)_Mob	0.415	-0.338	1.168	0.280
Mobility		0.548	-0.204	1.301	0.153
Walking speed	Ni (2014) TC_WS	0.059	-0.716	0.835	0.880
Walking speed	Gothe (2016)_WS	-0.366	-0.745	0.013	0.058
Walking speed	Oken (2006)_WS	0.167	-0.409	0.744	0.569
Walking speed		-0.130	-0.490	0.230	0.479

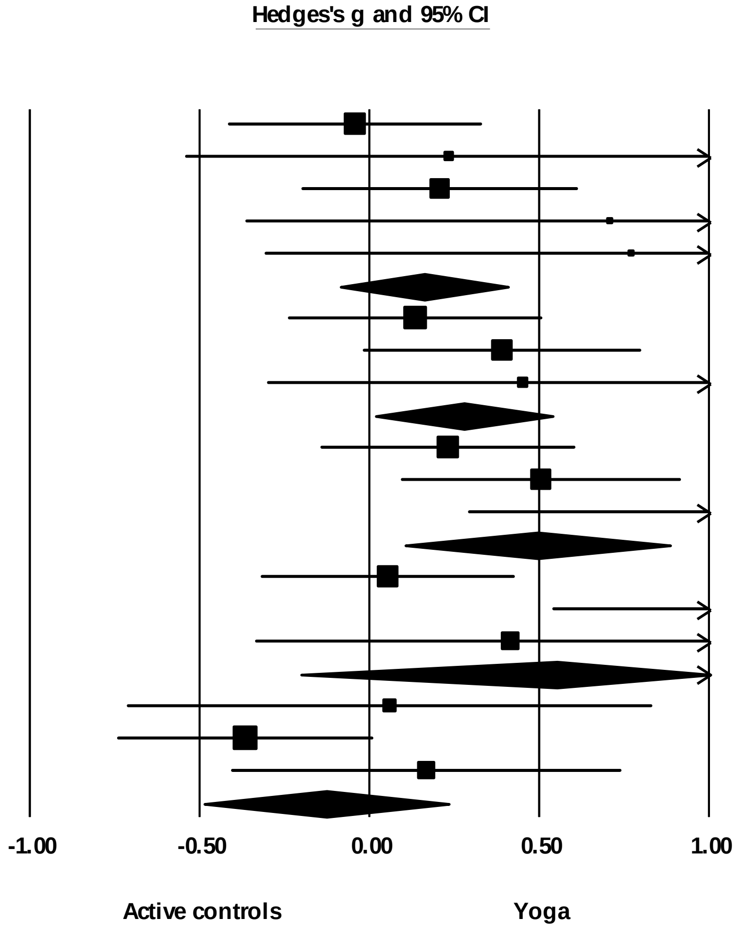

Bal: balance; TC: Tai Chi; Lflex: Lower body flexibility; LST: Lower limb strength; Mob: Mobility; WS: Walking speed

Heterogeneity data

Outcome	I square	P value
Balance	0	0.49
Lower body flexibility	0	0.59
Lower limb strength	47.44	0.15
Mobility	74.83	0.02
Walking speed	25.35	0.26

3. Manjunath (2005) [3]

Sensitivity analysis 1: Meta-analysis results (HRQoL outcome, sleep quality- yoga vs inactive controls) choosing yoga group and Ayurveda group (or herbal preparation) for Manjunath (2005)

Study name	Statistics for each study			
	Hedges's g	Lower limit	Upper limit	p-Value
Chen (2009)_Sleep	0.623	0.270	0.976	0.001
Chen (2010)1_Sleep	1.052	0.491	1.612	0.000
Hariprasad (2013)_Sleep	0.396	0.036	0.755	0.031
Manjunath (2005) Ay_Sleep	0.658	-0.072	1.388	0.077
	0.622	0.368	0.876	0.000

Hedges's g and 95% CI

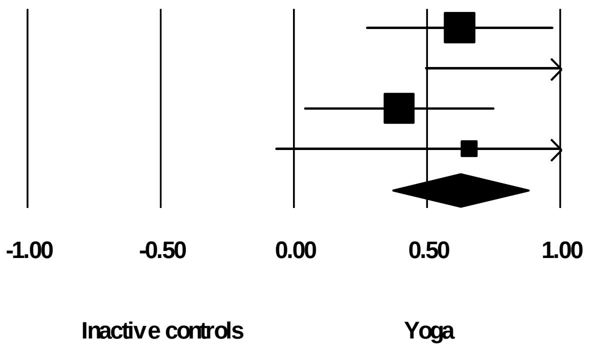

Sleep: Sleep quality; Ay: Ayurveda (herbal preparation)

Heterogeneity data

Outcome	I square	P value
Sleep	20.41	0.29

Sensitivity analysis 2: Meta-analysis results (HRQoL outcome, sleep quality- yoga vs inactive controls) choosing yoga group and wait-list group for Manjunath (2005)

Study name	Statistics for each study			
	Hedges's g	Lower limit	Upper limit	p-Value
Chen (2009)_Sleep	0.623	0.270	0.976	0.001
Chen (2010)1_Sleep	1.052	0.491	1.612	0.000
Hariprasad (2013)_Sleep	0.396	0.036	0.755	0.031
Manjunath (2005) WL_Sleep	1.026	0.361	1.691	0.002
	0.694	0.394	0.993	0.000

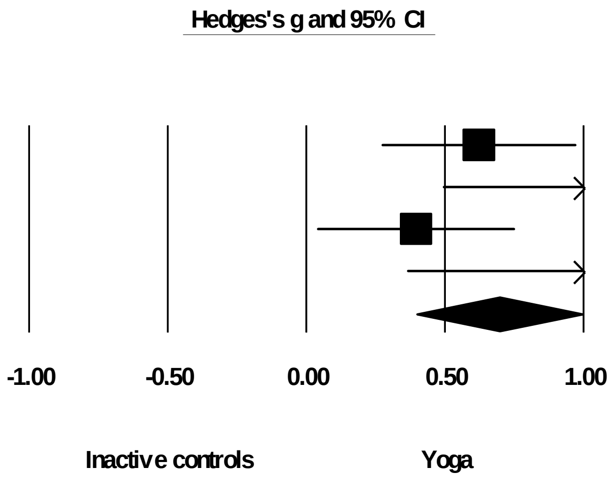

Sleep: Sleep quality; WL: Wait-list control

Heterogeneity data

Outcome	I square	P value
Sleep	41.69	0.16

4. **Krishnamurthy (2007)2 [4]**

Sensitivity analysis 1: Meta-analysis results (HRQoL outcome, depression- yoga vs inactive controls) choosing yoga group and Ayurveda group (or herbal preparation) for Krishnamurthy (2007)2

Outcome	Studyname	Statistics for each study			
		Hedges's g	Lower limit	Upper limit	p-Value
Depression	Bethany (2005)_Dep	1.228	0.326	2.130	0.008
Depression	Oken (2006)_Dep	0.171	-0.244	0.586	0.420
Depression	Wang (2010)_Dep	0.207	-0.713	1.126	0.659
Depression	Bonura (2014)_Dep	0.461	-0.026	0.948	0.064
Depression	Chen (2010)1_Dep	0.655	0.116	1.195	0.017
Depression	Chen (2009)_Dep	0.802	0.444	1.161	0.000
Depression	Noradechanunt (2017)_Dep	-0.120	-0.865	0.625	0.752
Depression	Krishnamurthy (2007)2 Ay_Dep	1.500	0.694	2.306	0.000
		0.582	0.263	0.900	0.000

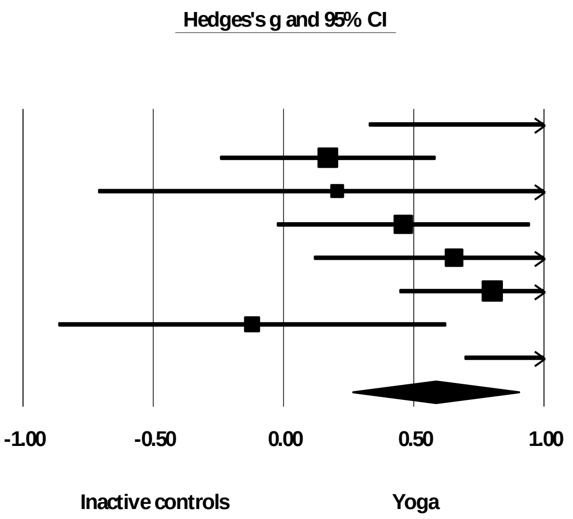

Dep: Depression; Ay: Ayurveda (herbal preparation)

Heterogeneity data

Outcome	I square	P value
Depression	57.61	0.02

Sensitivity analysis 2: Meta-analysis results (HRQoL outcome, depression- yoga vs inactive controls) choosing yoga group and waitlist control group for Krishnamurthy (2007)2

Outcome	Study name	Hedges's g	Lower limit	Upper limit	p-Value
Depression	Bethany (2005)_Dep	1.228	0.326	2.130	0.008
Depression	Oken (2006)_Dep	0.171	-0.244	0.586	0.420
Depression	Wang (2010)_Dep	0.207	-0.713	1.126	0.659
Depression	Bonura (2014)_Dep	0.461	-0.026	0.948	0.064
Depression	Chen (2010)1_Dep	0.655	0.116	1.195	0.017
Depression	Chen (2009)_Dep	0.802	0.444	1.161	0.000
Depression	Noradechanunt (2017)_Dep	-0.120	-0.865	0.625	0.752
Depression	Krishnamurthy (2007)2 WL_Dep	1.473	0.767	2.179	0.000
		0.593	0.268	0.918	0.000

Statistics for each study

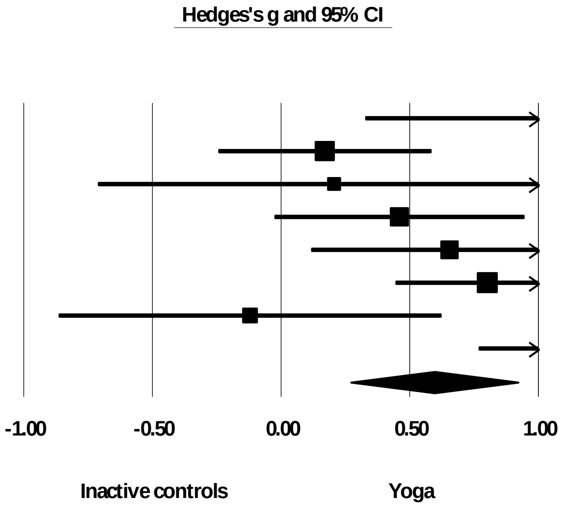

Dep: Depression; WL: Wait-list control

Heterogeneity data

Outcome	I square	P value
Depression	60.38	0.01

References

1. Bethany KH: **The effects of selected exercise modalities on stress, anxiety, and depression responses in the elderly.** *M.S.* The Florida State University, 2005.
2. Ni M, Mooney K, Balachandran A, Signorile JF: **Comparative impacts of Tai Chi, balance training, and a specially-designed yoga program on balance in older fallers.** *Archives of Physical Medicine and Rehabilitation* 2014, **95:**1620-1628.
3. Manjunath NK, Telles S: **Influence of Yoga & Ayurveda on self-rated sleep in a geriatic population.** *Indian Journal of Medical Research* 2005, **121:**683-690.
4. Krishnamurthy MN, Telles S: **Assessing depression following two ancient Indian interventions.** *Journal of Gerontological Nursing* 2007, **33:**17-23 17p.
