# Supplementary material for: The effects of yoga compared to active and inactive controls on physical function and health related quality of life in older adults- systematic review and meta-analysis of randomised controlled trials
Source: Int J Behav Nutr Phys Act. 2019 Apr 5;16:33. doi: 10.1186/s12966-019-0789-2 (PMC6451238; doi:10.1186/s12966-019-0789-2)
Supplement: Supplementary file 7 — Cluster randomisation adjustment results- Forest plots and homogeneity data. Results of the cluster randomisation adjustment analysis are provided in this document. This includes Forest plots and homogeneity data. (PDF 29 kb) [file 12966_2019_789_MOESM7_ESM.pdf]

Additional file 7. Cluster randomisation adjustment results- Forest plots and homogeneity data

Cluster randomisation adjustment analysis 1: Comparing yoga and inactive controls for physical function outcomes

Group by Outcome	Study name	Hedges's g	Lower limit	Upper limit	p-Value
		Statistics for each study			
Balance	Oken (2006)_Bal	0.169	-0.246	0.584	0.426
Balance	Wang (2010)_Bal	0.279	-0.939	1.498	0.653
Balance	Nick (2016) _Bal	2.062	1.296	2.829	0.000
Balance	Saravanakumar (2014)_Bal	0.823	-0.229	1.875	0.125
Balance	Tiedemann (2013)_Bal	0.402	-0.139	0.944	0.145
Balance	Morris (2008)_Bal	1.275	0.126	2.424	0.030
Balance	Tew (2017)_Bal	0.240	-0.327	0.807	0.407
Balance		0.700	0.185	1.215	0.008
Bodycomp	Chen (2008) M_BC	0.138	-0.451	0.727	0.646
Bodycomp	Chen (2008) SSY_BC	0.226	-0.372	0.824	0.459
Bodycomp	Tew (2017)_BC	-0.065	-0.631	0.500	0.822
Bodycomp	Bezerra (2014)_BC	0.146	-0.493	0.786	0.654
Bodycomp	Chen (2010)2_BC	0.287	-0.710	1.283	0.573
Bodycomp		0.120	-0.166	0.406	0.410
Lower body flexibility	Chen (2010)2_LFlex	0.679	-0.343	1.702	0.193
Lower body flexibility	Oken (2006)_LFlex	0.592	0.168	1.015	0.006
Lower body flexibility	Marques (2017)_LFlex	0.845	0.036	1.653	0.041
Lower body flexibility	Noradechanunt (2017)_Lflex	0.446	-0.309	1.200	0.247
Lower body flexibility	Chen (2008) M_Lflex	0.358	-0.235	0.951	0.236
Lower body flexibility	Chen (2008) SSY_Lflex	0.442	-0.161	1.045	0.151
Lower body flexibility	Wang (2010)_Lflex	-0.004	-0.995	0.986	0.993
Lower body flexibility	Tew (2017)_Lflex	0.418	-0.154	0.990	0.152
Lower body flexibility		0.489	0.263	0.716	0.000
Lower limb Strength	Oken (2006)_ST	0.386	-0.032	0.805	0.070
Lower limb Strength	Chen (2008) M_ST	0.185	-0.405	0.775	0.539
Lower limb Strength	Chen (2008) SSY_ST	0.678	0.065	1.290	0.030
Lower limb Strength	Noradechanunt (2017)_ST	0.987	0.196	1.779	0.014
Lower limb Strength	Tew (2017)_ST	-0.168	-0.734	0.399	0.562
Lower limb Strength	Tiedemann (2013)_ST	0.639	0.089	1.189	0.023
Lower limb Strength	Wang (2010)_ST	0.992	-0.096	2.079	0.074
Lower limb Strength	Leininger (2006)_ST	0.495	0.060	0.931	0.026
Lower limb Strength		0.449	0.216	0.682	0.000
Upper body flexibility	Vogler (2011)_Uflex	0.491	-0.141	1.123	0.128
Upper body flexibility	Chen (2010)2_Uflex	0.229	-0.765	1.224	0.651
Upper body flexibility	Noradechanunt (2017)_Uflex	0.327	-0.423	1.077	0.393
Upper body flexibility	Tew (2017)_Uflex	0.131	-0.435	0.697	0.650
Upper body flexibility		0.291	-0.053	0.636	0.098
Walking speed	Chen (2010)2_WS	0.441	-0.563	1.446	0.389
Walking speed	Chen (2008) M_WS	0.151	-0.438	0.741	0.614
Walking speed	Chen (2008) SSY_WS	0.933	0.306	1.560	0.004
Walking speed	Tiedemann (2013)_WS	0.973	0.406	1.541	0.001
Walking speed	Oken (2006)_WS	0.040	-0.522	0.603	0.888
Walking speed	Tew (2017)_WS	-0.310	-0.879	0.259	0.285
Walking speed		0.361	-0.080	0.802	0.109

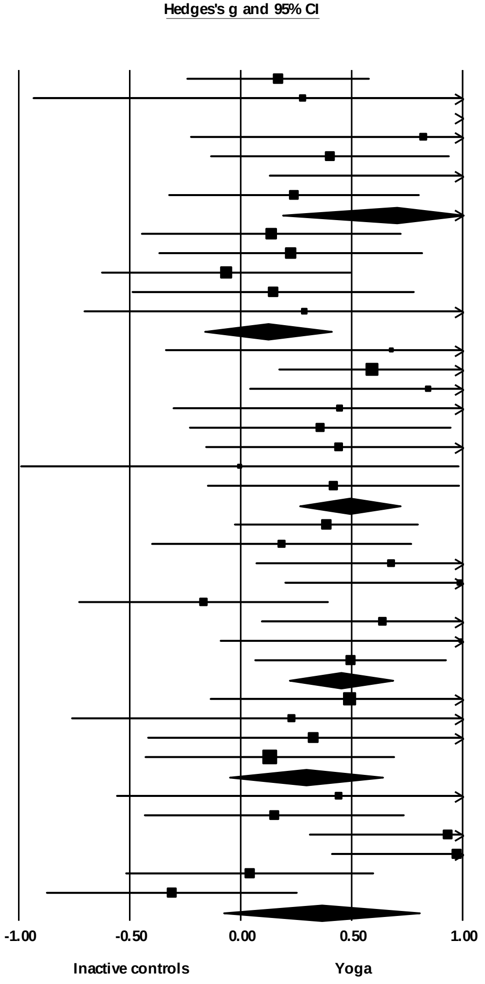

Bal: Balance; M: Yoga group with meditation; SSY: Short Silver Yoga; BC: Body composition; Lflex: Lower body flexibility; ST: Strength (Lower limb); Uflex: Upper body flexibility; WS: Walking speed

Heterogeneity data

Outcome	I-squared	P value
Balance	72.15	0.001
Body composition	0	0.96
Lower body flexibility	0	0.94
Lower limb strength	23.69	0.24
Upper body flexibility	0	0.87
Walking speed	66.13	0.01

Cluster randomisation adjustment analysis 2: Comparing yoga and inactive controls for HRQoL outcomes

Group by Outcome	Study name	Hedges's g	Lower limit	Upper limit	p-Value
		Statistics for each study			
Depression	Bethany (2005)_Dep	1.228	0.326	2.130	0.008
Depression	Oken (2006)_Dep	0.171	-0.244	0.586	0.420
Depression	Wang (2010)_Dep	0.207	-0.713	1.126	0.659
Depression	Bonura (2014)_Dep	0.461	-0.026	0.948	0.064
Depression	Chen (2010)1_Dep	0.608	-0.507	1.722	0.285
Depression	Chen (2009)_Dep	0.795	0.222	1.368	0.007
Depression	Noradechanunt (2017)_Dep	-0.120	-0.865	0.625	0.752
Depression	Krishnamurthy (2007)2 Ay_Dep	1.480	0.537	2.423	0.002
Depression	Krishnamurthy (2007)2 WL_Dep	1.403	0.557	2.248	0.001
Depression		0.628	0.278	0.978	0.000
Fear of falls	Morris (2008)_FOF	-0.185	-1.226	0.857	0.728
Fear of falls	Nick (2016)_FOF	1.197	0.527	1.867	0.000
Fear of falls	Tiedemann (2013)_FOF	0.058	-0.478	0.594	0.833
Fear of falls		0.393	-0.451	1.237	0.362
Per mental health	Vogler (2011)_PMH	1.383	0.687	2.079	0.000
Per mental health	Oken (2006)_PMH	0.085	-0.329	0.500	0.686
Per mental health	Chen (2009)_PMH	0.895	0.316	1.474	0.002
Per mental health	Bonura (2014)_PMH	0.443	-0.043	0.930	0.074
Per mental health	Wang (2010)_PMH	0.210	-0.709	1.130	0.654
Per mental health	Marques (2017)_PMH	0.067	-0.707	0.841	0.865
Per mental health	Noradechanunt (2017)_PMH	0.539	-0.219	1.298	0.164
Per mental health	Tew (2017)_PMH	0.719	0.136	1.303	0.016
Per mental health		0.538	0.229	0.848	0.001
Per physical health	Vogler (2011)_PPH	0.193	-0.431	0.817	0.544
Per physical health	Oken (2006)_PPH	0.519	0.098	0.940	0.016
Per physical health	Chen (2009)_PPH	0.961	0.378	1.544	0.001
Per physical health	Noradechanunt (2017)_PPH	0.032	-0.713	0.776	0.934
Per physical health		0.468	0.102	0.834	0.012
Sleep quality	Chen (2009)_Sleep	0.617	0.053	1.182	0.032
Sleep quality	Chen (2010)1_Sleep	0.971	-0.187	2.130	0.100
Sleep quality	Manjunath (2005) Ay_Sleep	0.620	-0.231	1.470	0.153
Sleep quality	Manjunath (2005) WL_Sleep	0.997	0.190	1.803	0.015
Sleep quality		0.742	0.359	1.126	0.000
Social health	Oken (2006)_Soc	0.157	-0.258	0.572	0.458
Social health	Wang (2010)_Soc	-0.296	-1.218	0.626	0.530
Social health		0.081	-0.297	0.459	0.675
Vitality	Leininger (2006)_Vit	0.248	-0.182	0.679	0.258
Vitality	Oken (2006)_Vit	0.310	-0.107	0.726	0.145
Vitality	Noradechanunt (2017)_Vit	0.518	-0.240	1.276	0.180
Vitality		0.312	0.034	0.591	0.028

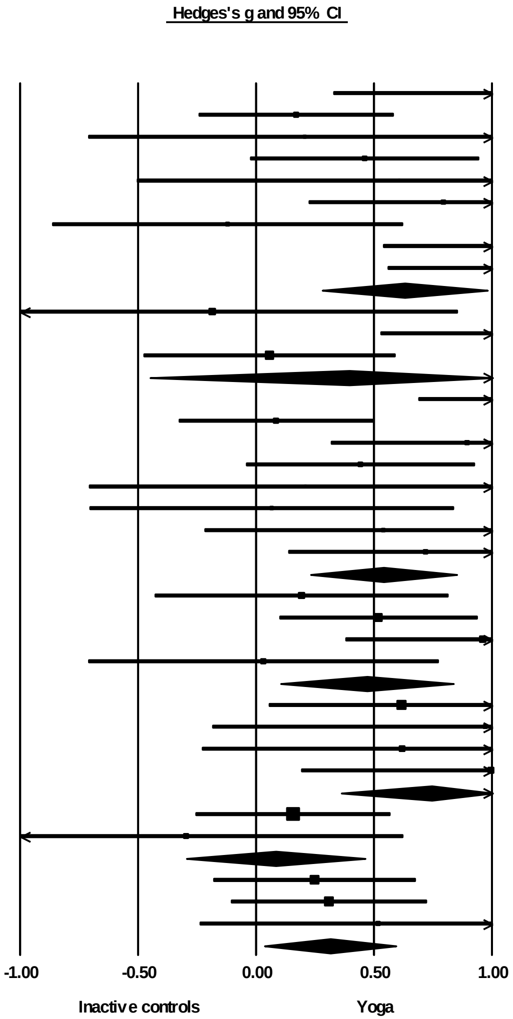

Dep: Depression; FOF: Fear of falls; Per mental health/PMH: Perceived mental health; Per physical health/PPH: Perceived physical health; Sleep: Sleep quality; Soc: Social health; Vit: Vitality; Ay: Ayurveda (herbal preparation); WL: Wait-list control

Heterogeneity data

Outcome	I-squared	P value
Depression	54.25	0.03
Fear of falls	75.64	0.02
Perceived mental health	50.03	0.05
Perceived physical health	38.17	0.18
Sleep quality	0	0.85
Social health	0	0.38
Vitality	0	0.83
